# "Is Cybermedicine Killing You?" - The Story of a Cochrane Disaster

**DOI:** 10.2196/jmir.7.2.e21

**Published:** 2005-06-30

**Authors:** Gunther Eysenbach, Per Egil Kummervold

**Affiliations:** ^2^Norwegian Centre for TelemedicineTromsøNorway; ^1^Centre for Global eHealth InnovationTorontoCanada

## Abstract

This editorial briefly reviews the series of unfortunate events that led to the publication, dissemination, and eventual retraction of a flawed Cochrane systematic review on interactive health communication applications (IHCAs), which was widely reported in the media with headlines such as "Internet Makes Us Sick," "Knowledge May Be Hazardous to Web Consumers' Health," "Too Much Advice Can Be Bad for Your Health," "Click to Get Sick?" and even "Is Cybermedicine Killing You?" While the media attention helped to speed up the identification of errors, leading to a retraction of the review after only 13 days, a paper published in this issue of JMIR by Rada shows that the retraction, in contrast to the original review, remained largely unnoticed by the public. We discuss the three flaws of the review, which include (1) data extraction and coding errors, (2) the pooling of heterogeneous studies, and (3) a problematic and ambiguous scope and, possibly, some overlooked studies. We then discuss "retraction ethics" for researchers, editors/publishers, and journalists. Researchers and editors should, in the case of retractions, match the aggressiveness of the original dissemination campaign if errors are detected. It is argued that researchers and their organizations may have an ethical obligation to track down journalists who reported stories on the basis of a flawed study and to specifically ask them to publish an article indicating the error. Journalists should respond to errors or retractions with reports that have the same prominence as the original story. Finally, we look at some of the lessons for the Cochrane Collaboration, which include (1) improving the peer-review system by routinely sending out pre-prints to authors of the original studies, (2) avoiding downplay of the magnitude of errors if they occur, (3) addressing the usability issues of RevMan, and (4) making critical articles such as retraction notices open access.

## A Series of Unfortunate Events

If you are interested in stories with happy endings, you would be better off reading some other story. In this story, not only is there no happy ending, there is no happy beginning and very few happy things in the middle.

-Lemony Snicket, A Series of Unfortunate Events

On October 16, 2004, three press releases from the University College London (UCL) (Multimedia Appendix 1), Wiley InterScience in the United Kingdom, publishers of The Cochrane Library, and the Center for the Advancement of Health (CFAH) in the United States were widely disseminated to announce the result of a just-published Cochrane review synthesizing "studies on Internet health" (UCL press release) or, more accurately, interactive health communication applications (IHCAs) [1]. The Cochrane review seemed to arrive at stunning results that "confound conventional wisdom" (quote of the Principal Investigator, taken from the UCL press release): the Cochrane investigators found that these applications lead to an increase in knowledge and positive feelings of social support, but they had deleterious effects on health outcomes, that is, "may leave [users] in worse health" (UCL press release). These surprising results were immediately jumped on by the mass media and led to widely publicized news stories around the globe, with often sensationalistic and oversimplified headlines, such as "Internet Makes Us Sick," "Knowledge May Be Hazardous to Web Consumers' Health," "Too Much Advice Can Be Bad for Your Health," "Click to Get Sick?" and even "Is Cybermedicine Killing You?" (see Figure 1, Table 1, and Multimedia Appendix 2).

**Figure 1:** A collage of headlines reporting on the IHCA Cochrane review

**Table 1:** Epidemic of misinformation: selected headlines from around the world reporting the results of the flawed Cochrane review, compiled in October 2004 (as of June 2005, most of these articles are still online, and not a single one carries a note on the retraction)

**Web Not Always Safe Health Source for Some** HON News (Health on the Net Foundation), Switzerland - Oct 21, 2004	**Internet medical advice risky** Big News Network.com, Australia - Oct 18, 2004
**Patients using the Net at risk: report** The Age, Australia - Oct 17, 2004	**Internet-based health information may be hazardous: study** CBC News, Canada - Oct 18, 2004
**Too Much Advice Could Be Bad for Your Health** HealthCentral.com - Oct 18, 2004	**Warning on internet health advice** Onlypunjab.com, India - Oct 18, 2004
**Too much information bad for your health, study shows** E-Health Insider, UK - Oct 18, 2004	**Click To Get Sick?** TIME - Oct 25, 2004
**Study: Internet Medical Advice Could Have Unintended Consequences** ihealthbeat, USA - Oct 18, 2004	**Internet makes us sick** The Times, UK - Oct 22, 2004
**Knowledge May be Hazardous to Web Consumers' Health** Newswise (press release) - Oct 17, 2004	**Warning on internet health advice** BBC News, UK - Oct 17, 2004
**Patient, don't try to heal thyself** DMeurope.com, Netherlands - Oct 19, 2004	**Beware of Internet health advises** Pravda, Russia - Oct 18, 2004
**Web Not Always Safe Health Source for Some** Forbes - Oct 21, 2004	**Warning over bad health advice online** Medical News Today, UK - Oct 18, 2004
**Logging on can make you sicker** ABC Science Online, Australia - Oct 19, 2004	**Fears over health 'cures' on the web** The Scotsman, UK - Oct 18, 2004
**Too much Internet advice is bad for your health: study** Canada.com, Canada - Oct 18, 2004

Few reporters seemed to have read the review, which actually did not speak about health websites or "the Internet" (as suggested in the press release and subsequent media reports) but of "Interactive Health Communication Applications (IHCAs)." According to the Cochrane review, the defining feature of an IHCA is "that it does not simply provide health information, but combines such information with at least one (and frequently more than one) additional service [such as] decision support, behaviour change support or peer support" [1], which excludes information-only websites. Ignoring this, the press release spoke of the "Internet" and contained statements like "knowledge-seekers become so steeped in information from the Internet they make treatment choices on their own, contradicting advice from their doctors." Many news outlets reprinted the press release verbatim, which also stated that "people who use their computers to find health information often wind up in worse condition than if they had listened to their doctor," or rephrased this into "Some people with chronic health problems who seek online advice would be better off just listening to their doctors." Some journalists even condensed this to "Patient, don't try to heal thyself." Statements like this—emphasizing that people should better listen to their doctor rather than going on the Web—made consumers, patients, self-care advocates, and eHealth experts wince, not only because they seemed factually questionable, but also because they were reminiscent of a dark pre-Internet era of paternalism and "doctor knows it all" mindsets, which many thought were long behind us [2-6].

Most eHealth researchers are driven by the belief that the Web and other interactive media applications play a major role in supporting patients with chronic conditions. At the same time, we are all for being on the cautious side, viewing eHealth applications with a critical eye, knowing that some people will not benefit from them, stressing that badly designed applications can harm patients, and monitoring unintended side effects and potentially negative outcomes [7]. Still, many eHealth researchers were surprised and angered by the sweeping and blatant comments stemming from this review, which seemed to ignore the growing literature on the effectiveness of many eHealth interventions, some of which have been published in this journal. Most researchers familiar with the literature know that the vast majority of such reports are actually positive—in fact, the proportion of positive studies is so overwhelming that it has been questioned whether negative studies are underreported [8].

**Figure 2:** The original (flawed) figure from the retracted Cochrane review [1], showing the reverted effect estimates favoring the control rather than IHCAs

The series of unfortunate events culminated when the scientific eHealth community debunked the Cochrane review as a "methodological disaster" [9]. Among several other flaws (outlined below), the review included severe extraction (or coding and data interpretation) errors leading to a complete reversal of the outcomes. Positive outcomes in the primary studies (such as reduction in encopresis [10]) were misinterpreted as negative (harmful) effects. As Per Egil Kummervold and colleagues listed in the feedback section of the Cochrane Library on October 28, 2004, at least 8 of 11 outcomes were reversed—letting the effect estimator appear on the left side ("favours control") instead on the right side ("favours intervention"). See Figure 2, which shows the flawed figure from the Cochrane review, and Multimedia Appendix 2, which shows corrections made by Kummervold et al.

These were stunning errors because anyone who read these primary reports could not possibly have come to the conclusion that any of these studies reported less favourable health outcomes in the IHCA groups. To date, it remains a mystery how respected and experienced investigators could arrive at these conclusions (unless investigators relied on research assistants or students to extract the data and did not bother to read the studies themselves, which is an unimaginable scenario for a Cochrane review). These errors were obviously magnified by aggressive marketing efforts of the investigators and publisher, who sent out three press releases that did not in any way caution readers about the results.

When the review was eventually retracted by the authors on October 29, 2004—only 13 days after the press release—the public hardly took notice. As illustrated in an article by Roy Rada in this issue of JMIR [11], the media remained quiet, too quiet. To date, many publications have not published any follow-up stories, and the impact will be long-lasting. As the Rada paper shows, the Web is still full of reports on the flawed Cochrane review, and Rada identified only one newspaper story about the retraction—the Canadian journalist said he found this out only by chance. (We are also aware of a report by Frith Rayner, published in the *Australian Doctor*, courtesy of Lee Ritterband.) The failure of the media to report the retraction has to do with either the fact that they simply did not know about it or with issues around how the media decides what will be newsworthy ("if it bleeds, it leads," "bad news are good news"). Another reason why it was not taken up could be that the press release reporting the retraction was not very clear in highlighting the magnitude of the error, and it contained little more than the message that the review was being reworked and that it was too early to say what the result would be. Few journalists would have understood that the errors invalidated the results completely, even reversed them.

Rada analyzes the impact of the review and draws a few lessons, some of which shall be complemented by this editorial, not least because the authors of this editorial were involved in the events that eventually led to a retraction.

## The Emperor Without Clothes

In a curious way, the media attention this review received—as detrimental as it was in sending out a false message to the public—also had a positive side in that it probably also sped up the identification of the errors. Without the media frenzy the results would possibly have remained unnoticed for a while, but with the worldwide media attention, peers quickly heard about the review.

For example, the editor of this journal (Gunther Eysenbach) was contacted by a journalist when the press release came out, was among the first who looked closely at the original review on October 16, and was one of the first who blew the whistle, pointing out that this emperor did not have clothes. Having read many of the primary studies that were pooled in the review, having done several systematic reviews in this area, and knowing the results of another review which JMIR published around the same time [12], he told the journalist who contacted him that the study seemed flawed. On October 25, he also posted a message on the Medical Webmasters Mailing List (MWM-L), where some researchers had started to discuss the study, warning readers not to take the review at face value [9].

Around the same time, on October 24, another researcher, Lee Ritterband—whose research was cited in the review and who had not seen a pre-print of it prior to publication—was alarmed by media reports and commented in a mailing list for Internet health intervention researchers: "While it is possible that some people may be worse off, we know that our interventions are quite effective, and this kind of fear-inducing 'findings' are the types of comments which our research, in part, must debunk." (Ritterband, personal communication, June 15, 2005). On the other side of the Atlantic, Per Egil Kummervold and colleagues at the Norwegian Centre for Telemedicine had also noticed that the numbers did not add up. They started a thorough investigation, reviewing the original data material, and on October 28 they notified the Cochrane Collaboration that the authors had made almost inconceivable mistakes, including reverting the direction of the results. On October 29, 2004, the review was retracted.

## The Three Principal Flaws

### Data Extraction and Coding Errors

The most devastating (and most obvious) error was the previously discussed blatant mistake of misinterpreting positive outcomes as negative outcomes (and vice versa). Kummervold's original list of errors is documented in Multimedia Appendix 2.

### Pooling Heterogeneous Studies

The second concern is that the studies were too heterogeneous to sensibly attempt a formal random effects meta-analysis using Cochrane's RevMan software. The resulting effect estimates are meaningless. The review guidelines of the Consumers and Communication Group state the following: "They [systematic reviewers] should also use caution when extracting and interpreting data, and when deciding whether to combine them statistically. Combining disparate data quantitatively may not always be appropriate, and qualitative synthesis may often be preferable" [13].

This is particularly true for pooling health outcomes, but equally problematic is to pool knowledge scores and behaviour change and social support measures from studies with interventions that had very little in common except that they were delivered electronically. Comparing different IHCAs against controls and pooling these results without paying any attention to the "ingredients" is like comparing all studies in which investigators used "blue pills" in the intervention arm—a pooling on the basis of the delivery mechanism rather than the "ingredient" is of limited value. And for every successfully IHCA-delivered intervention, one can probably find a similar intervention which is delivered on a badly designed IHCA, which does not mean that IHCAs per se are inappropriate delivery mechanisms.

A richer, deeper qualitative analysis to answer questions like "what seem to be the success factors in terms of how the intervention and the trial should be designed" would have been more appropriate and more informative. Qualitative synthesis prevents us from drowning in a river that on average is only 3 feet deep.

As an aside, while the authors focused on extracting outcome measures which could support their postulated pathway of action, it would have been very informative to extract and report attrition rates as secondary outcomes (ie, the percentage of users who dropped out and/or did not use the application), not only because nonuse of the application may explain a lack of an effect, but also because such data from numerous studies could be useful in identifying some of the factors (predictors) for nonuse/dropout as postulated in the "Law of Attrition" [14]. In health informatics, issues around adoption are at least as important as health outcomes [15], and systematic reviews in this area should try to extract and synthesize adoption measures.

### Scope and Lack of Comprehensiveness

The third problem with the IHCA Cochrane review, which has not yet been discussed on the Cochrane feedback section, is the scope of the review and the lack of truly comprehensive searches within the scope the authors defined. The scope may be too broad in some respect (making the review unmanageable and confusing by lumping together too many different applications), and too narrow in others (eg, by excluding pure patient-doctor or peer-to-peer communication, or by focusing on chronic diseases).

In particular, the authors decided to exclude electronic decision aids and computer/Internet-delivered cognitive behavioural therapy (CBT) programs. High-quality CBT applications, such as ODIN (Overcoming Depression on the InterNet), were not cited in the review [16]. This creates a considerable bias, as CBT applications are among the most successful interventions. These important exclusions were neither mentioned in the press release nor in any media reports.

There is also some confusion about the scope of the review, in particular, whether applications that provide social support by enabling peer-to-peer communication were included. The original definition of the Science Panel on Interactive Communication and Health [17] of the term "IHC application" is as follows:

[IHCAs are] the operational software programs or modules that interface with the end user. This includes health information and support Websites and clinical decision-support and risk assessment software (which may or may not be online), but does not include applications that focus exclusively on administrative, financial, or clinical data, such as electronic medical records, dedicated clinical telemedicine applications, or expert clinical decision-support systems for providers.

However, the Cochrane review team narrowed this definition by only including studies on applications that, apart from delivering health information, had another component (eg, decision support, peer-to-peer support), thereby excluding simple information-only websites. The fact that simple health information sites were not in the scope of the review was not communicated properly in the press release and was widely misunderstood by the media. Journalists reported, for example, that "the study found no evidence that Web health information [*sic*] helps people with chronic diseases" (*HealthDayNews*) or that "people who use their computer to find out more about their condition end up in worse health than those who do not" (*The Times*). While the Cochrane investigators may have had good reasons to exclude simple health information websites, there was a remarkable divergence between what the public understood the review was about and the actual inclusion criteria. Leaving aside all other flaws, such as coding errors, it appears problematic to issue press releases that suggest that the Internet is harmful when the actual review excluded things like websites, Internet-based CBT programs, and possibly even peer-to-peer support groups.

On the latter point, the definition of IHCA used by the Cochrane review team leaves considerable ambiguity about whether or not "pure" peer-to-peer groups are in the scope of the review—and ambiguities at the protocol stage are often a recipe for disaster [18]. One may argue that peer-to-peer support on the Internet is always embedded in a wealth of health information on the Web and would therefore meet the definition and fall within the scope of this review.

A final concern is that a comparison with another systematic review [19] suggests that the searches were less than comprehensive or that the reference screening process was sloppy. In a systematic review on applications with peer-to-peer components [19] (which was not cited in the Cochrane review), 20 randomized controlled trials of IHCAs (all of which had peer-to-peer components) were identified. Of these, only 6 studies were included in the Cochrane review, 3 were excluded, but more than half (as many as 11 studies [20-29]) were not cited or mentioned in the Cochrane review, although many of them appear relevant or should at least have been explicitly excluded (see Multimedia Appendix 3). While it is admittedly difficult (or impossible) to find all relevant papers in this area, the fact that more than half of the studies from a previously published systematic review were not cited is disturbing.

## What the Various Parties Should Learn From This

### Retraction Ethics

In the "publication ethics" literature, which deals with scientific misconduct such as duplicate publication, underreporting, and authorship issues, there is remarkably little discussion on how retractions due to error or misconduct should be handled by investigators and the media. In the case discussed here, the Cochrane Collaboration and the investigators were, as the Rada paper [11] shows, not very effective in getting word of the retraction out to the public. Obviously, there are also very little incentives for the investigators' organization or the publisher/editor to blare out an embarrassing error with the same vigour as the original report. One may argue that it is a matter of ethics to try to match the aggressiveness of the original dissemination campaign if errors are detected and a wrong story needs to be corrected. Researchers and their organizations may have an ethical obligation to track down journalists who reported the misinformation and to specifically ask them to publish an article correcting the error.

Similarly, in our view there is an ethical duty for journalists to respond to such requests and to react to reports on errors or retractions with stories that have the same prominence as the original story. In other words, if the original report was worth a space on the title page, the retraction should be reported on the same prominent spot. In cases for which it is possible to change or add something to the original story (online articles), this should also be done.

### Responsibility of the Cochrane Collaboration

#### No Systemic Failures?

The Cochrane press release that was issued when the original report was retracted contained the following statement: "The Cochrane Collaboration regrets that this particular review was found to contain inaccuracies, apologises unreservedly, has acted swiftly to mitigate both this error (which arose from individual error and not systemic failures) and the likelihood of it being repeated, and undertakes to ensure that the corrected results are published as soon as possible" (Cochrane press release).

What is interesting here is that it took the Cochrane Collaboration only a few days to determine that there were no "systemic failures," which, in our view, is questionable. Perhaps a better approach would have been to set up an independent group to analyze the mistakes made and to wait for them to come back with some recommendations, rather than swiftly dismissing any possibilities for systemic errors.

#### Failure of the Pre-Publication Peer-Review System

One remarkable and obvious "systemic" problem seems to be the apparent total failure of the pre-publication peer-review system. Most eHealth researchers (and certainly those whose work was cited in the review) state that it took them only minutes to figure out that something was wrong with the review, which suggests that the 4 peer reviewers who reviewed the manuscript were not intimately familiar with the work done in this area. One potential policy change that the Cochrane Collaboration may have to make is a *requirement* to invite authors of the primary studies to comment on the systematic review, a sort of semi-open peer review. Rada suggests making the peer review completely open to the public, which is another consideration. One could for example use pre-print servers [30] to post drafts of reviews before they are published. However, this would diminish the newsworthiness of such reports [31] and, due to the Ingelfinger rule, may prevent such reports from being published in other academic journals [32].

#### Has the Magnitude of the Errors Been Downplayed?

As noted above, the Cochrane Collaboration and the investigators have not been successful in getting the word out about the error in a timely manner. It is not sufficient to wait for a corrected version to appear (which was promised for April 2005), hoping that the media and the public will remember the original story and correct their impressions of it. The press release issued by Cochrane seems to downplay the severity of the errors. It does not say that the errors were so grave that they literally led to a reversal of the conclusions, even though it was clear to any informed observer that the initial message Cochrane disseminated was the 180-degree opposite of what should have been reported.

The admittance of an error was half-hearted, and the marketers at Cochrane tried to use even the retraction press release as an opportunity to emphasize how good Cochrane reviews are compared with non-Cochrane reviews: "It has been demonstrated that Cochrane Systematic Reviews are of comparable or better quality and are updated more often than the reviews published in print journals" [33]. It would have been wiser in this situation to cite a paper with a very similar focus [12], which happened to appear in this journal (JMIR), rather than citing a paper that suggests that reviews developed outside of Cochrane are usually of worse quality, even though in this case the situation was exactly the opposite.

#### Usability of RevMan

Another issue Cochrane should carefully look at is the usability of RevMan, the software used to support meta-analyses. From the experience of one of the authors (GE), RevMan clearly has some usability issues, most notably that it is far too easy to accidentally "flip" the direction of outcomes. This may have been a contributing factor to the errors in this case. The principal investigator wrote in the Cochrane Communication Consumer and Communication Group newsletter that "RevMan has a mind of its own and I don't think I could have managed it without our very own IT whizzkid,...the lead research fellow on the review" [34]. If software is so difficult to use that it takes an "IT whizzkid" to enter the data (as opposed to the medical experts who understand the primary papers), errors seem to be pre-programmed.

#### At Least Retractions Should Be Open Access!

The Cochrane Library is (amazingly) still not an Open Access publication. This may have been a contributing factor to why the retraction remained largely unnoticed by the public and many fellow researchers. The UCL press release (Multimedia Appendix 1) refers readers to the Wiley website, which is subscription-access only. Even the "Reason for Withdrawal" cannot be accessed by nonsubscribers (as of May 30, 2005). Shouldn't at least retraction statements be made open access, and shouldn't this be a standard practice across all toll-access journals?

**Figure 3:** The "Reason for Withdrawal" behind closed doors—only subscribers have the privilege of learning about the retraction (as of May 30, 2005)

## The Damage Done

Fortunately, this particular Cochrane review warning patients to abstain from a specific type of intervention was not about a drug or other clinical intervention, whose withdrawal could have cost lives.

But damage was done: statements in the press release suggesting that "patients are better off listening to their doctor than going to the Internet" have outraged patient advocates (rightly so) and eroded the public's trust in the medical profession, which appeared to warn of the dangers of the Internet for selfish reasons. This was expressed in a posting by a patient on the BrainTalk forum, who wrote, "If the medical profession had its way this forum would be illegal" [35].

The myth of the Internet causing harm to your health may be here to stay, at least for a while, and policy makers and researchers searching the Web for evidence on the effectiveness of IHCAs will inevitably run into media remnants of the Cochrane review and cite it without bothering to read the original or corrected version. While Rada [11] failed to find any citation to the review in the Web of Science database (which is not surprising since in most traditional journals [not JMIR] it takes many months or years from submission to publication), one of the authors (GE) has already seen, as a peer reviewer, one book chapter and one thesis citing the Cochrane review without mentioning the retraction status.

It is our hope that by publishing this editorial and the Rada paper we do our part in making the public and the research community aware of this series of unfortunate events. While much of the damage created in this case is irreversible, lessons should be learnt so that future disasters can be avoided.

## Multimedia Appendix 1

UCL press release

## Multimedia Appendix 2

Errors in outcomes

## Multimedia Appendix 3

Potentially missed studies

## Multimedia Appendix 4

Media responses

## Abbreviations

IHCA: interactive health communication application
