# Supplementary material for: "Is Cybermedicine Killing You?" - The Story of a Cochrane Disaster
Source: J Med Internet Res. 2005 Jun 30;7(2):e21. doi: 10.2196/jmir.7.2.e21 (PMC1550649; doi:10.2196/jmir.7.2.e21)
Supplement: Supplementary file 1 [file jmir_v7i2e21_app1.pdf]

Multimedia Appendix 1

UCL Press Release http://www.ucl.ac.uk/media/archive/archive-release/?internethealth

(with retraction notice as added by UCL)

!Please Note!

The review "Interactive Health Communication Applications for people with chronic disease" by Elizabeth Murray, Jo Burns, Sharon See Tai, Rosalind Lai, Irwin Nazareth, published on issue 4 2004 of The Cochrane Library , has been found to contain an error. This meant the direction of change was incorrect for several clinical and behavioural outcomes.

The authors and editorial team have responded immediately, and will be withdrawing the review from The Cochrane Library for revision to correct the error.  The authors and editorial team apologise to all concerned.

An official statement has been posted here:
http://www.mrw.interscience.wiley.com/cochrane/clsysrev/articles/CD004274/frame.html

Please do not use information from the following release until the review has been re-analysed

18 October 2004

# Knowledge may be hazardous to web consumers' health

People who use their computers to find information about their chronic disease often wind up in worse condition than if they had listened to their doctor, according to a University College London (UCL) review of studies on internet health.

Using interactive computer tools does improve the medical knowledge of people with diabetes, asthma or other chronic conditions, and does provide them with positive feelings of social support, according to researchers reviewing 28 randomized controlled trials involving 4,042 participants. But there was no evidence that cyber-medicine helps people change their behavior and startling evidence that it may leave them in worse health.

"This whole finding confounds conventional wisdom," says lead author Dr Elizabeth Murray of UCL's Department of Primary Care and Population Sciences. The authors looked at studies that measured the effectiveness of Interactive Health Communication Applications (IHCAs) on people's information gain, feelings of social support, self-efficacy, behavior change and overall clinical outcomes.

IHCAs had a positive effect on people's information gain and feelings of social support; no effect on self-efficacy (the belief that behavior change is possible) or on actual behavior change; and a strikingly negative effect on outcomes.

Dr Murray said there are two possible reasons for the paradox between active knowledge-seekers and their seemingly worsening health.

One reason may be that when they learn of small, but important, statistical effects of a treatment they become less frightened and thus unmotivated to change the way they might if a doctor bluntly told a person with diabetes to control her sugar or face death. "But actually," Murray explains, "if you become more knowledgeable you realize it's all rather a long way off. They are less frightened and that reduces their motivation to be really strict in their control."

A second reason might be because knowledge-seekers become so steeped in information from the Internet they make treatment choices on their own, contradicting advice from their doctors. For instance, a diabetic person might be told by a doctor to lower blood sugar but decide, based on his own interpretation of data, that the short-term tradeoffs of not complying are worth the long-term risks.

"We found that people who use these things (IHCAs) had more sugar in their blood than those who didn't," Dr Murray said.

The researchers cannot explain the finding that the interventions had no effect on behavior change but nevertheless resulted in worse outcomes. Murray and her team conclude that more research is needed to fully understand the negative effect of interactive health applications on clinical outcomes and whether some computer health programs can be designed to improve them.

The review appears in the October issue of the Cochrane Collaboration, an international organization that evaluates medical research. Systematic reviews draw evidence-based conclusions about medical practice after considering both the content and quality of existing medical trials on a topic.

In this review, the definition of an Interactive Health Communication Application was a computer-based information source combined with one or more additional services, such an on-line support group, chat room or tailored advice based on data provided by the user. Murray says, however, that some researchers "worry that the friends you make on computer are not right sort of friends, won't be there for you and may not be good for your social well being."

The authors suggest that other assumptions about interactive health care are flawed. For example, they say, "If knowledge was all that was needed to promote healthy behavior, smoking would not be as prevalent as it is." Further, well-informed health consumers may not, as expected, drive down the use of health care but may increase it by demanding specific and possibly more costly treatments.

The review bolstered previous conclusions that computer-assisted learning is at least as effective, if not more so, than traditional methods of conveying information. But the past studies did not yield evidence favoring or opposing computer health information as a means of achieving behavior change.

The authors caution that the results of this review are more important for policy rather than for practice, particularly in Western, highly developed health care systems although they should not yet be viewed as a method of cost-containment.

Notes to the editor

For more information or to set up an interview please contact Jenny Gimpel at the UCL Media Relations Office on +44 (0)20 7679 9739, e-mail j.gimpel@ucl.ac.uk.

Alternatively, for US queries please contact the Health Behavior News Service on +1 (202) 387-2829 or www.hbns.org .

The Cochrane Collaboration is an international non-profit and independent organization that produces and disseminates systematic reviews of healthcare interventions and promotes the search for evidence in the form of clinical trials and other studies of interventions. Visit http://www.cochrane.org for more information.
