# Supplementary material for: "Is Cybermedicine Killing You?" - The Story of a Cochrane Disaster
Source: J Med Internet Res. 2005 Jun 30;7(2):e21. doi: 10.2196/jmir.7.2.e21 (PMC1550649; doi:10.2196/jmir.7.2.e21)
Supplement: Supplementary file 2 [file jmir_v7i2e21_app2.pdf]

Overview IHCA vs Control. Clinical outcomes

Per Egil Kummervold, Norwegian Centre for Telemedicine
Martin Jensen, Norwegian Centre for Telemedicine
Per Hjortdahl, University of Oslo

- Exact numbers in Fig. 05 p 76 of Murray 2004.
- Complete references p26 Murray 2004.

**Reference**	Bartholomew 2000
**Measurement**	Functional status
**Description**	Bartholomew (Table 2, p 275) reports increased functional status. Interpreted correctly.
**Conclusion**	OK

**Reference**	Flatley-Brennan 1998
**Measurement**	Health status
**Description**	The numbers seems to be from Table 4, p. 501: "Differential decline in health status". Interpreted correctly.
**Conclusion**	OK

**Reference**	Brown 1997
**Measurement**	HbA1c
**Description**	Brown (table 1, p 86) reports an increase in HbA1c of 0.86 for intervention group(SD=1.64), and an increase of 0.66 for control group (SD=1.89). This should be reported as a negative effect for intervention group.
**Conclusion**	Error

**Reference**	Glascow 2003
**Measurement**	HbA1c
**Description**	Glascow (Table 3, p 417) reports a reduction in mean Alc hemoglobin from 7.54 to 7.42 (-0.12) for intervention group (SD=1.68) and an increase for control group (SD=1.56) from 7.35 to 7.68 (+0.33). This equals the numbers reported by Murray. This should however be interpreted as a positive effect in favour of intervention.
**Conclusion**	Error

**Reference**	Gustavson 1999
**Measurement**	Physical function
**Description**	We have not yet been able to determine how Murray has calculated the change in "physical function", as baseline is not reported in the article. Some of the numbers are reported in Table 1 p 4. Murray probably has more data. There is very little/no difference between the groups, so this is of no significance.
**Conclusion**	Unconclusive

**Reference**	Krishna 2003
**Measurement**	Days with Asthma Symptoms
**Description**	Krishna (Table 4, p. 507) reports a reduction for intervention group (SD=113.3) in mean days with astma symptoms of -80.6 and a reduction for control group (SD=109.0) of 49.6. This should however be interpreted as a positive health effect for intervention group.
**Conclusion**	Error

**Reference**	Lehmann 2003
**Measurement**	HbA1c
**Description**	Lehrmann (Table 4A p 14) reports a reduction HbA1c of 0.8 for intervention group, and 0.1 for control group. This should be interpreted as a postivie result for intervention group.
**Conclusion**	Error

**Reference**	Ritterband 2003
**Measurement**	Bowel Accidents per week
**Description**	Ritterband's abstract is positive in favour of intervention group. In figure 2 it looks like the reduction in "bowel accidents" is largest for intervention group. Unfortunately our copy makes it impossible to read the exact numbers. It is however obvious that this is positive clinical effect in favour of intervention group.
**Conclusion**	Error

**Reference**	Turnin 1992
**Measurement**	HbA1c
**Description**	Turnin reports a significant reduction in HbA1c for intervention group in abstract. In the text it is reported a reduction in HbA1c level in the intervention group of -0.6, and an increase in mean HbA1c of 0.2 in the control group. This should be interpreted as a positive clinical effect for intervention group.
**Conclusion**	Error

**Reference**	Turnin 2001
**Measurement**	Cholestrol
**Description**	Turnin reports a reduction in Cholesterol level for intervention group of -0.31, and a reduction for control group of -0.35. This should be interpreted as a negative effect for intervention group.
**Conclusion**	Error

**Reference**	Whylie-Rosett 2001
**Measurement**	BMI
**Description**	Whylie-Rosett's (Table 2, p 1159) reports mean reduction of BMI for intervention group of 0.8, and reduction for control group of 0.4, This should be reported as a positive effect for intervention group.
**Conclusion**	Error
