# Supplementary material for: "Is Cybermedicine Killing You?" - The Story of a Cochrane Disaster
Source: J Med Internet Res. 2005 Jun 30;7(2):e21. doi: 10.2196/jmir.7.2.e21 (PMC1550649; doi:10.2196/jmir.7.2.e21)
Supplement: Supplementary file 3 [file jmir_v7i2e21_app3.pdf]

Multimedia Appendix 3 – Missed Studies

This Table is a subset of "Online Table 1" from Eysenbach et al. systematic review on electronic peer-to-peer support [1], which was published several months before the release of the Cochrane Review. While this systematic review also contained non-randomized studies, we only list the studies which were classified as RCT (excluding Holden's n-of-1 studies with STARBRIGHT).
As most of the p2p systems were embedded into more complex applications, they should have been identified and at least screened for inclusion/ exclusion criteria by the IHCA research team. However, the Cochrane review on IHCA failed to cite 11/20 RCT's listed here.

Online Table 1: Interventions

Intervention short	Ref-ID (Author, Year, first pg)	Included/ Excluded/ Not mentioned	Health condition	Intervention description
TalkNet, Care Mail etc	Alemi1996-10[2]	not mentioned	drug users	Complex intervention includes TalkNet (voice bulletin board system, available to only some participants), but also care mail (a voice mail service for patients and providers), community health rap (a service for answering patients questions at home), announcements (a service for sending motivational messages and prayers to patients), conference calls (a service that enables patients to share insights with each other), and follow-up (an automated method of monitoring patients in the community). Subjects received health education, access to pediatric and prenatal providers, testimonials, and weekly prayers.
D-Net	Barrera2002[3]-McKay2002[4]	incl	type 2 diabetes	4 arms. (1) Internet information only (control); (2) virtual community: information plus peer directed moderated forum, topic-related bulletin board, live chat rooms, information; (3) information plus personal self-management coach and access to an Internet blood glucose tracking and graphing system allowing to enter their blood glucose levels.(4) Combined condition (all 1, 2 and 3). All participants received home computers and training.
ComputerLink Alzheimer	Bass1998-20[5]-Casper1995[6]-Brennan1995[7]-McClendon1998-403[8]	incl	caregivers of alzheimer's disease sufferers	ComputerLink Alzheimer. Caregivers in the experimental group received computer terminals, with access to ComputerLink, a specialized computer network developed to provide systematic assistance to Alzheimer's caregivers, includes a) Communication: un-moderated bulletin board, e-mail, and anonymous question & answers to a nurse moderator, b) Information: electronic encyclopedia, and c) decision support.

Online Table 1: Interventions

Intervention short	Ref-ID (Author, Year, first pg)	Included/ Excluded/ Not mentioned	Health condition	Intervention description
ComputerLink AIDS	Brennan1998-489[9]	incl	AIDS	ComputerLink AIDS: Equipment provided and access given to private computer network that includes a) Communication: unmoderated bulletin board and nurse-moderated question & answers bulleting board, e-mail b) Information: electronic encyclopedia, and c) decision support.
Student Bodies Internet	Celio2000-650[10]	not mentioned	healthy body weight	3 arms: (1) Wait-list control; (2) Student Bodies (Internet), a structured 8-wk cognitive-behavioral intervention, includes face to face sessions and a moderated Internet discussion group, mandatory and optional weekly assignment, e-mail follow up and feedback, access to self help and psycho-educational materials (text, audio- and video presentations, Internet self-monitoring journals, and behavior change exercises); (3) academic face to face classroom course (Body Traps). Participants (students) received academic credit for completion.
D-Net	Glasgow2003-410[11]	incl	type 2 diabetes	3 arms: (1) Internet information only (control); (2) information plus peer support - including moderated forum, topic-related bulletin board, and live chatroom ; (3) information plus personal self-management coach
CHESS AIDS	Gustafson1999-1[12]	incl	AIDS	CHESS (Comprehensive Health Enhancement Support System), AIDS module, installed on PC, provides facilitated Internet discussion groups, instant library (full text articles), FAQ, self-assessment questions, personal stories, referral directory, decision support, and private communication with professionals and other patients.
CHESS Breast Cancer	Gustafson2001-435[13]	incl	breast cancer	CHESS (Comprehensive Health Enhancement Support System), Breast Cancer module, installed on PCs with modem. Brief training (1 hr.) provided, user manual left with participant. Consisted of 11 computer-based services integrated to provide information (Q&A, library, consumer guide, referral directory, decision-making tools (assessment, health charts, decision aid, action plan), and support services (discussion groups via bulletin boards, Ask and Expert, and personal stories.

Online Table 1: Interventions

Intervention short	Ref-ID (Author, Year, first pg)	Included/ Excluded/ Not mentioned	Health condition	Intervention description
Weight Loss Program HB	Harvey-Berino2002-103[14]	not mentioned	weight loss	3 arms: Initial 15 wk weight loss treatment program given to all groups prior to randomization. Then randomized into (1) control group (no treatment); (2) Therapist Led: face-to-face group therapy with self-monitoring (paper) diaries telephonic follow up, self-monitoring data (calorie intake, exercise expenditure, body weight) via postcards); (3) Internet support: facilitated bi-weekly Internet chat sessions, self monitoring data, video with explanation of topics for discussion, e-mail among peers, bulletin board.
Weight Loss Program HB	Harvey-Berino2002-1254[15]	not mentioned	weight loss	3 arms: Initial 24 wk weight loss treatment program given to all groups prior to randomization. Then randomized into (1) control group (no treatment); (2) frequent in-person support, therapist led face-to-face group therapy; (3) facilitated bi-weekly Internet chat sessions, self-monitoring data, video of the group therapist who introduced a topic for discussion, e-mail among peers, bulletin board.
WebCT	Johs-Artisensi2002-thesis[16]	not mentioned	smoking	Smoking cessation brochures plus access to a Web site (WebCT) that included a forum, chat rooms, links to Internet pharmacies, links to quitting tools, FAQ, Internet and motivational wallpapers to download.
mailing list, book, video	Lorig2002-792[17]	not mentioned	back pain	Closed, moderated, e- mail discussion group, book and videotape about back pain.
D-Net	McKay2001-1328[18]	excl	type 2 diabetes	"D-Net Active Lives Intervention", 8-wk online personalized physical activity program from personal coach, plus peer support from electronic support group conference area. Web site also features private personal database (activity diary) and library of articles on physical activity
Smoking Compuserve	Schneider1990-141[19]	not mentioned	smoking	Smoking cessation system on CompuServe, with similar features as previous EIES (Electronic Information Exchange System) versions. Allows access to a "stop smoking forum", allowing to read comments of other smokers and to enter own comments, although authors say that "participants also occasionally engaged in real time discussions" (without further explanation). This was a 4-arm trial (full version/shortened version of the system, with/without forum),. The system contained a 4-week interactive, personalized stop-smoking behavior modification program (tailored instructions on the basis of responses to questions), smoking diary, access to "help with urges" messages, and a "group progress graph"

Online Table 1: Interventions				
**Intervention short**	**Ref-ID (Author, Year, first pg)**	**Included/ Excluded/ Not mentioned**	**Health condition**	**Intervention description**
Weight Loss Program Tate	Tate2001-1172[20]	excl	weight loss	Structured Internet-delivered behavioral weight loss program including a sequence of 24 weekly lessons, weekly submission of self-monitoring diaries, weekly email recommendations from a therapist, opportunity to ask questions to the therapist, and the opportunity for social support among group members through a discussion forum. Also access to resource on Web site as described for the control group.
Weight Loss Program Tate	Tate2003-1833[21]	excl	weight loss	Both groups (control and intervention) had access to (separate) peer-to-peer message boards [in the context of this review, before-after data from the control group are of interest]. Intervention group received behavioral e-counseling, face to face behavioral sessions, peer-to-peer bulletin board, access to study website for weight loss information, self-monitoring electronic diary, personalized feedback from therapist by e-mail.
Student Bodies CD-ROM	Winzelberg1998-339[22]-Winzelberg1998-thesis[23]	not mentioned	healthy body weight	Student Bodies intervention, consisting of two components: (1) a moderated email support group. Members participated anonymously, (2) Student Bodies software on CD-ROM, providing a psycho-educational intervention. The Student Bodies software (on CD-ROM) contains audio- and video presentations and provides a psycho-educational intervention, addressing body image dissatisfaction, excessive weight concerns, and dieting or restrained eating patterns.
Student Bodies Internet	Winzelberg2000-346[24] Dev1999-510[25]	not mentioned	healthy body weight	Student bodies (redesigned version from the 1998 trial, now focusing on body image), delivered through the Internet, consisting of 1) online discussion group providing a forum for participants, now integrated in the Web-program (previously email-based) 2) structured Body Image program
Bosom Bodies	Winzelberg2003-1164[26]	not mentioned	breast cancer	Bosom Buddies, a 12-week, moderated, web-based psycho-educational support group (weekly readings sent by e-mail. Web site also offers personal journal. In addition, WebTV given to patients with no prior Internet access.
Student Bodies Internet	Zabinski2001-401[27]	not mentioned	healthy body weight	Student bodies (Internet program) consisting of 1) moderated online discussion group providing a forum for participants 2) Student Bodies software with psycho-educational material for eating disorders.

Reference List

1. Eysenbach G, Powell J, Englesakis M, Rizo C, Stern A. Health related virtual communities and electronic support groups: systematic review of the effects of online peer to peer interactions. *BMJ* 2004;**328**:1166.

2. Alemi F, Stephens RC, Javalghi RG, Dyches H, Butts J, Ghadiri A. A randomized trial of a telecommunications network for pregnant women who use cocaine. *Med.Care* 1996;**34**:OS10-OS20.

3. Barrera M, Jr., Glasgow RE, McKay HG, Boles SM, Feil EG. Do Internet-based support interventions change perceptions of social support?: An experimental trial of approaches for supporting diabetes self-management. *Am J Community Psychol* 2002;**30**:637-54.

4. McKay HG, Glasgow RE, Feil EG, Boles SM, Barrera JM. Internet-based diabetes self-management and support: Initial outcomes from the diabetes network project. *Rehabilitation Psychology* 2002;**47**:31-48.

5. Bass DM, McClendon MJ, Brennan PF, McCarthy C. The buffering effect of a computer support network on caregiver strain. *Journal of Aging & Health.* 1998;**10**:20-43.

6. Casper GR, Calvitti A, Brennan PF, Overholt JL. ComputerLink: the impact of a computer network on Alzheimer's caregivers' decision-making confidence and skill. *Medinfo* 1995;**8 Pt 2**:1546.

7. Brennan PF, Moore SM, Smyth KA. The effects of a special computer network on caregivers of persons with Alzheimer's disease. *Nurs Res* 1995;**44**:166-72.

8. McClendon MJ, Bass DM, Brennan PF, McCarthy C. A computer network for Alzheimer's caregivers and use of support group services. *Journal of Mental Health & Aging* 1998;**4**:403-20.

9. Flatley-Brennan P. Computer network home care demonstration: a randomized trial in persons living with AIDS. *Comput Biol Med* 1998;**28**:489-508.

10. Celio AA, Winzelberg AJ, Wilfley DE, Eppstein-Herald D, Springer EA, Dev P *et al*. Reducing risk factors for eating disorders: comparison of an Internet- and a classroom-delivered psychoeducational program. *J Consult Clin Psychol.* 2000;**68**:650-7.

11. Glasgow RE, Boles SM, McKay HG, Feil EG, Barrera M. The D-Net diabetes self-management program: long-term implementation, outcomes, and generalization results. *Prev Med* 2003;**36**:410-9.

12. Gustafson DH, Hawkins R, Boberg E, Pingree S, Serlin RE, Graziano F *et al*. Impact of a patient-centered, computer-based health information/support system. *Am J Prev Med* 1999;**16**:1-9.

13. Gustafson DH, Hawkins R, Pingree S, McTavish F, Arora NK, Mendenhall J *et al*. Effect of computer support on younger women with breast cancer. *J.Gen.Intern.Med.* 2001;**16**:435-45.

14. Harvey-Berino J, Pintauro SJ, Gold EC. The feasibility of using Internet support for the maintenance of weight loss. *Behav Modif* 2002;**26**:103-16.

15. Harvey-Berino J, Pintauro S, Buzzell P, DiGiulio M, Casey GB, Moldovan C *et al*. Does using the Internet facilitate the maintenance of weight loss? *International Journal of Obesity & Related Metabolic Disorders: Journal of the International Association for the Study of Obesity.* 2002;**26**:1254-60.

16. Johs-Artisensi, Jennifer Lynn. The effect of web-based support as an adjunct to a self- help smoking cessation program. 121. 2002. University of North Texas.
Ref Type: Thesis/Dissertation

17. Lorig KR, Laurent DD, Deyo RA, Marnell ME, Minor MA, Ritter PL. Can a Back Pain E-mail Discussion Group Improve Health Status and Lower Health Care Costs?: A Randomized Study. *Arch Intern Med* 2002;**162**:792-6.

18. McKay HG, King D, Eakin EG, Seeley JR, Glasgow RE. The diabetes network internet-based physical activity intervention: a randomized pilot study. *Diabetes Care* 2001;**24**:1328-34.

19. Schneider SJ, Walter R, O'Donnell R. Computerized Communication as a Medium for Behavioral Smoking Cessation Treatment: Controlled Evaluation. *Comp Hum Behav* 1990;**6**:141-51.

20. Tate DF, Wing RR, Winett RA. Using Internet technology to deliver a behavioral weight loss program. *JAMA* 2001;**285**:1172-7.

21. Tate DF, Jackvony EH, Wing RR. Effects of Internet behavioral counseling on weight loss in adults at risk for type 2 diabetes: A randomized trial.
12. *JAMA: Journal of the American Medical Association* 2003;**Vol 289**:1833-6.

22. Winzelberg AJ, Taylor CB, Sharpe T, Eldredge KL, Dev P, Constantinou PS. Evaluation of a computer-mediated eating disorder intervention program. *Int J Eat Disord* 1998;**24**:339-49.

23. Winzelberg, Andrew James. Evaluation of a computer-mediated eating disorder prevention program. 126. 1998. Stanford University.
Ref Type: Thesis/Dissertation

24. Winzelberg AJ, Eppstein D, Eldredge KL, Wilfley D, Dasmahapatra R, Dev P *et al*. Effectiveness of an Internet-based program for reducing risk factors for eating disorders. *J Consult Clin Psychol* 2000;**68**:346-50.

25. Dev P, Winzelberg AJ, Celio A, Taylor CB. Student bodies: psycho-education communities on the web. *Proceedings / AMIA ...Annual Symposium.* 1999;510-4.

26. Winzelberg AJ, Classen C, Alpers GW, Roberts H, Koopman C, Adams RE *et al*. Evaluation of an internet support group for women with primary breast cancer. *Cancer* 2003;**97**:1164-73.

27. Zabinski MF, Pung MA, Wilfley DE, Eppstein DL, Winzelberg AJ, Celio A *et al*. Reducing risk factors for eating disorders: targeting at-risk women with a computerized psychoeducational program. *International Journal of Eating Disorders.* 2001;**29**:401-8.
