# Supplementary material for: "Is Cybermedicine Killing You?" - The Story of a Cochrane Disaster
Source: J Med Internet Res. 2005 Jun 30;7(2):e21. doi: 10.2196/jmir.7.2.e21 (PMC1550649; doi:10.2196/jmir.7.2.e21)
Supplement: Supplementary file 4 [file jmir_v7i2e21_app4.pdf]

Macmillan cancer relief County Durham Appeal
NEWSQUEST (North East) Ltd A Gannett Company

Is cyber medicine killing you?

Barry Nelson
22/10/2004

While the Internet can make us into instant experts on our health, a new report suggests that a little knowledge can be a dangerous thing. Health correspondent BARRY NELSON reports.

PATIENTS using the Internet to find out information about chronic disease rather than listening to their doctor could be putting their health at risk, according to researchers. Using interactive computer tools, such as online support groups and chatrooms, does help improve the knowledge of people with conditions such as asthma and diabetes and provides positive feelings of social support. But researchers from University College London have come to the conclusion that there was no evidence that "cyber medicine" helped people change their behaviour.

They even say there is evidence that using interactive services may actually leave patients in worse health.

The team, led by Dr Elizabeth Murray from the Department of Primary Care and Population Sciences, reviewed 28 trials involving more than 4,000 participants to measure the effectiveness of Interactive Health Communication Applications (IHCAs).

These were defined as a computerbased information sources combined with one or more additional services, such as online support groups, chatrooms or tailored advice based on data provided by the user.

The researchers found that the IHCAs had a positive effect on people gaining information and feelings of social support. But they concluded that the applications had no effect on making patients believe that changing their behaviour for the better was possible or in actually resulting in behaviour change.

They also said that they had a "strikingly negative effect" on health outcomes, leaving some in worse health.

Dr Murray says there were a number of reasons why there was an apparent contradiction between actively seeking knowledge and patients' seemingly worse health. One reason was that when patients learn of small but important statistical effects of their treatment, they become less frightened.

[illegible]

KNOWLEDGE MAY BE HAZARDOUS TO WEB CONSUMERS' HEALTH - Mozilla Firefox
File Edit View Go Bookmarks Tools Help
http://www.cfah.org/hbns/news/internet10-18-04.cfm

Release Date: Oct. 18, 2004

KNOWLEDGE MAY BE HAZARDOUS TO WEB CONSUMERS' HEALTH

By Ira Allen, Contributing Writer
Health Behavior News Service

A data analysis error by the authors of the review published by the Cochrane Library may change the conclusion of the article upon which this story is based. PLEASE DO NOT USE THIS STORY UNTIL THE REVIEW IS RE-ANALYZED. For further background, go to http://www.update-software.com/ccng/ccng.exe?SourceID=CD004274

People who use their computers to find information about their chronic disease often wind up in worse condition than if they had listened to their doctor, according to a new review of studies on Internet health.

Using interactive computer tools does improve the medical knowledge of people with diabetes, asthma or other chronic conditions, and does provide them with positive feelings of social support, according to researchers reviewing 28 randomized controlled trials involving 4,042 participants. But there was no evidence that cyber-medicine help people change their behavior and startling evidence that it may leave them in worse health.

"This whole finding confounds conventional wisdom," says lead author Elizabeth Murray, M.D., Ph.D., of the University College Medical School in London. The authors looked at studies that measured the effectiveness of "Interactive Health Communication Applications" on people's information gain, feelings of social support, "self-efficacy," behavior change and overall clinical outcomes.

IHCAs had a positive effect on people's information gain and feelings of social support; no effect on "self-efficacy" (the belief that behavior change is possible") or on actual behavior change; and a strikingly negative effect on outcomes.

Murray said there are two possible reasons for the paradox between active knowledge-seekers and their seemingly worsening health.

One reason may be that when they learn of small, but important, statistical effects of a treatment they become less frightened and thus unmotivated to change the way they might if a doctor bluntly told a person with diabetes to control her sugar or face death. "But actually," Murray explains, "if you become more knowledgeable you realize it's all rather a long way off. They are less frightened and that reduces their motivation to be really strict in their control."

A second reason might be because knowledge-seekers become so steeped in information from the Internet they make treatment choices on their own, contradicting advice from their doctors. For instance, a diabetic person might be told by a doctor to lower blood sugar but decide, based on his own

Done
start
6:03 AM Saturday 28/05/2005

HON - News : Web Not Always Safe Health Source for Some - Mozilla Firefox

http://www.hon.ch/News/HSN/521830.html

HON News

Health On the Net Foundation

Search news on 300 medical topics and themes

All Web sites | HONcode sites | HONselect | News | Conferences | Images

Themes: A B C D E F G H I J K L M N O P Q R S T U V W X Y Z

Browse archive:
2005: M A M F J
2004: D N O S A J J M A M F J
2003: D N O S A J J M A M F J

Web Not Always Safe Health Source for Some

Doesn't help people with diabetes and other chronic diseases change behavior

Other news for:
Diabetes Mellitus
Physicians
Asthma
Resources from HONselect

THURSDAY, Oct. 21 (HealthDayNews) -- Internet-based health information may be hazardous to people with chronic diseases such as diabetes or asthma, say University College London researchers.

They reviewed 28 studies on health information from the Web and found that using interactive computer tools does help people improve their medical knowledge and provides them with positive feelings of social support.

But the study found no evidence that Web health information helps people with chronic diseases make behavior changes that will benefit them. In fact, people who use computers to access information about their disease often end up in worse condition than if they had listened to their doctor.

"This whole finding confounds conventional wisdom," study author Dr. Elizabeth Murray, of the department of primary care and population sciences, said in a prepared statement.

She said patients who access a lot of Web-based health information may decide to make their own treatment choices, even if those decisions are contrary to a doctor's advice.

For example, a person with diabetes may be told by a doctor to lower blood sugar. But, based on his or her own interpretation of data from the Internet, the patient may decide the short-term tradeoffs of not controlling blood sugar are worth the long-term risks.

Murray added that increased knowledge about their disease and treatment may also make people less frightened and less motivated to be strict about their control of diabetes or other chronic diseases.

The study appears in the October issue of the *Cochrane Collaboration* .

More information

The U.S. National Institutes of Health has information about **healthy Web surfing** .

SOURCE: University College London, news release, Oct. 17, 2004

Copyright © 2004 **ScoutNews LLC** . All rights reserved.

Resources from HONselect: HONselect is the HON's medical search engine. It retrieves scientific articles, images, conferences and web sites on the selected subject.

Chronic Disease	Diabetes Mellitus	Delivery of Health Care
Health Resources	Universities	Therapeutics
Blood Glucose	Computers	Cooperative Behavior
Science	Behavior	Blood

Find: deposit Find Next Find Previous Highlight Match case

Done

7:41 AM Monday 30/05/2005

www.timesonline.co.uk
TIMES ONLINE
The best of The Times and The Sunday Times, in real time

From city apartments to country manors

Health

October 23, 2004

Body&Soul: Stories behind the news

Internet makes us sick

BY SIMON CROMPTON

Does surfing for health information do you more harm than good?

HEALTH websites are second only to porn pages in popularity and many doctors have viewed internet information as similarly corrupting.

But, just as there are signs that doctors are beginning to embrace our thirst for health knowledge as a force for good, a new study defies all our expectations.

An authoritative review by the Department of Primary Care and Population Sciences, at University College London, has come to the conclusion that people who use their computer to find out more about their condition end up in worse health than those who do not.

Elizabeth Murray found that people with chronic conditions such as diabetes and asthma who accessed computer resources did benefit in some ways — their medical knowledge improved and they felt supported. But when it came to actually changing their behaviour to make themselves more healthy, they failed miserably. What's more, they ended up in worse health than those who didn't consult the internet at all.

This wasn't meant to happen. Conventionally, knowledge-seekers are believed to benefit from better health.

Dr Murray thinks there could be two explanations. Either it's because the more learned we are, the less frightened we become and less motivated to make lifestyle changes. Or we become so confused by conflicting information we get from doctors and the internet that we make our own, sometimes [illegible]

There is nothing like time away to rejuvenate the body, relaxation, activity or exploration. **Order a FREE brochure**

SPECIAL

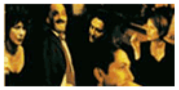

French Film Café
The votes are cast, discover the winner of our search for the ultimate French film. Claim your free tickets

ADVERTISEMENT

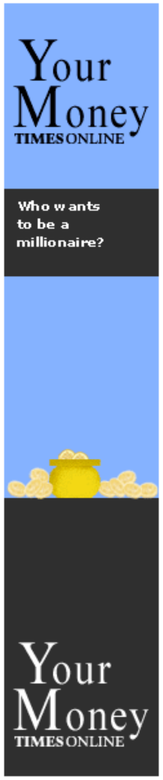

LATEST
Free bets
Visit Sports Book for our special betting offers
Travel search
Search our database of holiday ideas home and [illegible]

iHealthBeat.org - Mozilla Firefox
File Edit View Go Bookmarks Tools Help
http://www.ihealthbeat.org/index.cfm?Action=dspItem&itemID=106418 Go beat murray cochrane
~AdSense ~blue ~BMJ ~CNN ~dict ~EasyWeb ~Globe ~Google Scholar ~HubMed ~iplanet ~JMIR ~mls.ca ~PayPal ~PubMed ~SPIEGEL TOStar
Bowker's Books In Print… | Open Journal Systems:… | Google Search: quotes … | Open Journal Systems:… | Journal of Medical Inter… | iHealthBeat.org | Google Search: Global …

Features
- Today's News
- Commentary
- Interviews
- Multimedia
- Data Points
- Upcoming Events
- CHCF Highlights

Upcoming Events
- Get Connected Knowledge Forum
  June 27 - 29 | Grapevine, Texas
- NCVHS Full Committee Meeting
  June 29 - 30 | Washington, D.C.
- Ask the Experts: Health Information Technology
  June 30 | webcast
- Summer Institute in Nursing Informatics
  July 20 - 23 | Baltimore

Past Issues

Select Date

Services
- Login
- Advanced Search
- Help
- Site Map
- Glossary
- Contact Us

Welcome! Visitors wishing to view more than two items will be asked to login or register.

forward next article

Public Health & Research

Study: Internet Medical Advice Could Have Unintended Consequences

October 19, 2004

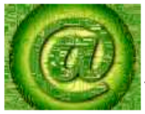

People with chronic illnesses who take medical advice from the Internet or computer programs could end up in worse health than if they follow their physicians' orders, according to a recent University College of London study, BBC News reports. However, the study also found that interactive technology can improve patients' medical knowledge (*BBC News*, 10/17).

The study looked at the effect of Interactive Health Communication Applications, or computer-based information sources that include services such as Internet support groups or chat rooms, on 4,042 adults and children with long-term conditions, such as diabetes or asthma.

The study found that the Internet did help patients understand their illnesses better and find support from others with the same conditions. However, there is no evidence that it helps patients change their behavior, according to the study, the *Ottawa Citizen* reports. For example, computer tools did not help diabetics make better food choices (Spears, *Ottawa Citizen*, 10/18). "We found that people who use these things had more sugar in their blood than those who didn't," lead researcher Dr. Elizabeth Murray said (*BBC News*, 10/17). Also, asthmatics who used the Internet to find health information had less control over their condition (*Ottawa Citizen*, 10/18).

The findings, which were published in the Cochrane Collaboration this month, are "surprising" because they go against the popular belief that well-informed patients will be healthier than those who are less informed, the London *Daily Telegraph* reports (Hall, London *Daily Telegraph*, 10/18). Murray proposed two reasons for the patterns. First, after doing independent research, patients become less frightened of their conditions, so they might not feel the same sense of urgency they get from a physician. Second, patients who conduct their own research might choose their own treatment and ignore their physicians' instructions (*Ottawa Citizen*, 10/18).

Murray called for physicians and politicians to consider the consequences of patients choosing to ignore their physicians' advice (*Daily Telegraph*, 10/18).

back next article

forward

Related Stories
- Study: Online Health Info Doesn't Replace Docs' Advice
  05/13/2004
- Online Health Seekers Torn Between Doc, Web as Best Info Source
  03/18/2004
- Report: Consumers Need Better Access to Reliable Online Health Info
  02/13/2004
- Study: Online Info Can Improve Patient Decisions, Save Time
  01/20/2004
- More Americans Going Online for Health Info
  01/14/2004

Too much information bad for your health, study shows - Microsoft Internet Explorer
File Edit View Favorites Tools Help

E-Health Insider
Tel: +44 (0)20 8871 5258 | Email: info@e-health-media.com
NEWSLETTER SIGN-UP 27 Jun 2005
your email address here GO

21st century systems for primary care
the phoenix partnership
CISCO SYSTEMS
THIS IS THE POWER OF THE NETWORK now

ADD A COMMENT SEND TO A FRIEND PRINT

Too much information bad for your health, study shows

18 Oct 2004

A study published by University College London has found that people suffering from chronic illnesses such as diabetes or asthma who use software or the internet to get information can actually end up damaging their own health.

The review, which appears in the latest issue of the Cochrane Collaboration, found that in one instance, diabetics who followed healthcare advice from the internet had more sugar in their blood than those that didn't.

"This whole finding confounds conventional wisdom," said Dr Elizabeth Murray, head author of the study.

One reason suggested for this is when people see unfiltered information about, for instance, effects of the treatment of diabetes, without it being put into perspective it discourages them from moderating their behaviour to control their sugar. "If you become more knowledgeable, you realise it's all rather a long way off. They are less frightened and that reduces their motivation to be really strict in their control." Another could be that advice on the internet could superficially contradict that of clinicans.

Furthermore, just because good advice was available, says Dr Murray, doesn't mean that people will follow it. "If knowledge is all that was needed to promote healthy behaviour, smoking would not be as prevalent as it is," said the report.

Another problem that healthcare providers could suffer is that people who have been informed of different ways their illness could be treated might demand to receive the least cost-effective treatments available.

Dr Murray said that more research was needed to fully study the negative effects the flood of information available over the internet has on people's health, and how health websites can be structured to counter this. Nevertheless, the study did conclude that computer-based learning was effective and that this could be harnessed.

The Cochrane Collaboration is an international organisation that uses evidence-based, systematic reviews to evaluate existing medical research about topics, and draws conclusions based on the quality of the information available.

Â© 2004 E-HEALTH-MEDIA LTD. ALL RIGHTS RESERVED.

FEATURED RECRUITERS
ElanIT Health
Guided SOLUTIONS LIMITED
ideal recruitment
SOFTWARE OF EXCELLENCE
Pactum Recruiting Professionals IT • Engineering • Science

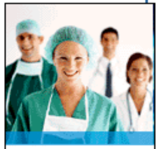

meeting the challenges of healthcare systems implementation
BestTreatments Click here for better health decisions
E-HEALTH INSIDER SPONSORED FEATURE E-Learning

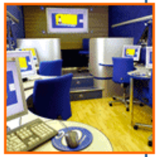

Learn HL7 & SNOMED Abies the ladder to success
InterSystems CACHE Powering the world's most
Done

Warning over bad health advice online - Microsoft Internet Explorer

File Edit View Favorites Tools Help

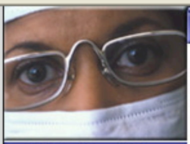

Medical News Today

home XML feed

News Categories -->

view news categories

Health Professionals

- Respiratory
- Breast Cancer
- GI
- Cardiovascular
- Psychiatry
- Prostate Cancer
- UK Learning Resources
- Medicines Interactive

Patient Resources

- Migraine
- Heartburn
- Blood Pressure
- Asthma

Other Links

- Latest Medical News
- News Archive Search
- About Us
- Advertising
- Medical Newsletter
- RSS/XML News Feed
- JavaScript News Feed
- Medical Toolbar
- Your Opinions
- Links
- Contact Us

Tell a Friend

Your Name:

Your E-mail:

News Archive: Search

Tuesday 28th June

10 Rules For Fat loss
Learn the 10 Idiot Proof Rules of Fat Loss & Lose 9 lbs in 11 days.

Give Time By Volunteering
Help The Community You Live In. It's Easy, Become A Volunteer Today.

Ads by Goooooogle

Medical Abbreviations

Go!

www.medilexicon.com

< back to previous page | < back to news headlines | sign up to our newsletter

Warning over bad health advice online

18 Oct 2004

email this to a friend | printer friendly page | view all opinions on this | write an opinion on this

Following health advice given on the Internet could leave people in worse health, a new study has warned.

The research from University College London (UCL) has shown that many people would be in a better state of health if they followed advice from their doctor rather than relying on information found online.

Although the UCL team found that using the Internet improves people's knowledge of medical conditions, the evidence suggests that so-called "cyber-medicine" does not help people change their behaviour.

One explanation could be that the more people know about a disease, the less concerned they become about the effect it will have on them.

People are less frightened when they discover more about an illness such as diabetes, because they realise there is no immediate threat and are therefore not motivated to change their habits.

Lead researcher Elizabeth Murray suggested people could be making their own decisions as to how they should best treat an illness based on information they have found over the Internet, ignoring their doctor's advice.

Such an approach has led to many people actually being worse off than if they had not found out anything about their

condition.

http://www.hda-online.org.uk

For more information on diabetes click here.

Huge database of hospitals world wide .

UNICEF has mobilized aid in India, Indonesia, Malaysia, the Maldives, Thailand and Sri Lanka.

Water supplies, health kits, tents, school supplies and more.

DONATE NOW TO PROTECT CHILDREN AT RISK

unicefusa.org

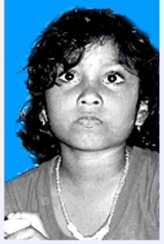

Ads by Goooooogle

Discuss Health Problem.
Free, anonymous, unbiased, & nonprofit health discussion forum

Value Of Internet Health Advice Questioned - Mozilla Firefox
File Edit View Go Bookmarks Tools Help
http://www.informatics.nhs.uk/cgi-bin/item.cgi?id=929

Value Of Internet Health Advice Questioned

A **University College London** study suggests that many people with chronic disease should not rely on the internet for health advice.

The study found interactive computer tools do improve medical knowledge, but found no evidence that they change behaviour positively. In fact, some evidence suggested that they left people in worse health.

The UCL team reviewed the effect of interactive web tools - known technically as Interactive Health Communication Applications (IHCAs) - on people with long-term conditions such as diabetes and asthma.

In total they looked at 28 studies covering 4,042 participants.

Lead researcher Dr Elizabeth Murray said she was surprised that the tools seemed to boost medical knowledge, but, if anything, have a negative effect on the users' health.

Dr Murray said one reason for this apparent paradox may be that titbits of information may lull users into a false sense of security.

Thus they become less motivated to control their condition in the way they would be if faced with a blunt instruction from a doctor.

Alternatively, users may become so steeped in information that they make treatment choices on their own, contradicting advice from their doctors.

For instance, a diabetic person might be told by a doctor to lower blood sugar but decide, based on his own interpretation of data, that the short-term tradeoffs of not complying are worth the long-term risks.

Dr Murray said: "We found that people who use these things had more sugar in their blood than those who didn't."

The researchers say more work is needed to fully understand the negative effect of interactive health applications on clinical outcomes - and whether some computer health programs can be designed to improve them.

The UCL team defined an IHCA as a computer-based information source combined with one or more additional services, such as an on-line support group, chat room or tailored advice based on data provided by the user.

Search
Visitor Register Now
Saturday May 28 2005
Login / Logout

Related news

Health informatics research

- BCS Summer Conference on Health Informatics
  5th-Jul
- Health Care Informatics Symposium
  28th-Jun
- Advance Notice of National Health Informatics Week
  8th-Apr
- Research Opportunities Boosted By Electronic Records
  30th-Mar
- Accolade Winners Announced
  24th-Mar
- Clinical Decision Support Systems Backed In Review
  16th-Mar
- US Report Backs Digitally Advanced Hospitals
  11th-Mar
- Computers In Healthcare Can Improve Decision Making
  9th-Mar
- Telemedicine Not Integrated In Routine Healthcare
  7th-Mar
- Computers Can Reduce Errors And Improve Patient Safety
  1st-Mar

Informatics & direct patient care

Done
start
5:44 AM Saturday 28/05/2005

HealthOrbit, Inc. - News Details - Mozilla Firefox

File Edit View Go Bookmarks Tools Help

http://healthorbit.ca/NewsDetail.asp?opt=1&nltid=014181004

~AdSense ~blue ~BMJ ~CNN ~dict ~EasyWeb ~Globe ~Google Scholar ~HubMed ~iplanet ~JMIR ~mls.ca ~PayPal ~PubMed ~SPIEGEL TOStar

Open Journal Systems: Sub... | HealthOrbit, Inc. - News... | EasyWeb | PayPal - My Account | The Beach Tour - Beaches ... | Brochures

Treatment Guidelines ODG Treatment in Workers Comp based on empirical medical evidence

Positive Thinking - Women Do you have a negative chatterbox inside your head? Let us help.

Reflective Happiness Plan A series of online exercises to improve your emotional well-being.

Employee Awards Terryberry - Employee Rewards Motivation, retention, incentives.

Ads by Goooooogle

UCL

Knowledge may be hazardous to web consumers' health

18 October 2004 -- People who use their computers to find information about their chronic disease often wind up in worse condition than if they had listened to their doctor, according to a University College London (UCL) review of studies on internet health.

Using interactive computer tools does improve the medical knowledge of people with diabetes, asthma or other chronic conditions, and does provide them with positive feelings of social support, according to researchers reviewing 28 randomized controlled trials involving 4,042 participants. But there was no evidence that cyber-medicine helps people change their behavior and startling evidence that it may leave them in worse health.

"This whole finding confounds conventional wisdom," says lead author Dr Elizabeth Murray of UCL's Department of Primary Care and Population Sciences. The authors looked at studies that measured the effectiveness of Interactive Health Communication Applications (IHCAs) on people's information gain, feelings of social support, self-efficacy, behavior change and overall clinical outcomes.

IHCAs had a positive effect on people's information gain and feelings of social support; no effect on self-efficacy (the belief that behavior change is possible) or on actual behavior change; and a strikingly negative effect on outcomes.

Dr Murray said there are two possible reasons for the paradox between active knowledge-seekers and their seemingly worsening health.

One reason may be that when they learn of small, but important, statistical effects of a treatment they become less frightened and thus unmotivated to change the way they might if a doctor bluntly told a person with diabetes to control her sugar or face death. "But actually," Murray explains, "if you become more knowledgeable you realize it's all rather a long way off. They are less frightened and that reduces their motivation to be really strict in their control."

A second reason might be because knowledge-seekers become so steeped in information from the Internet they make treatment choices on their own, contradicting advice from their doctors. For instance, a diabetic person [illegible]

Transferring data from healthorbit.ca...

start | Desktop | Links | 98% | 6:03 AM Saturday 28/05/2005

People who use their computers to find health information often wind up in worse condition than if they had listened to their doctor - Mozilla Firefox

File Edit View Go Bookmarks Tools Help

http://www.news-medical.net/?id=5621

~AdSense ~blue ~BMJ ~CNN ~dict ~EasyWeb ~Globe ~Google Scholar ~HubMed ~iplanet ~JMIR ~mls.ca ~PayPal ~PubMed ~SPIEGEL TOStar

Open Journal Systems: Sub... | People who use their co... | EasyWeb | PayPal - My Account | The Beach Tour - Beaches ... | Brochures

News-Medical.Net...

Medical News

Materials Nanotechnology Building AZoNetwork

Medical News Categories

Ads by Google
Medical News
Health Care News
Diabetes
Clinical Trials

- Home Page
- NEW Medical Newsletter
- Institutions A to Z
- Medical A to Z
- Express News Submit
- Child Health News
- Disease/Infection News
- Devices/Technology
- Healthcare News
- Medical Condition News
- Medical Procedure News
- Medical Patent News
- Medical Research News
- Medical Study News
- Men's Health News
- Medical Science News
- Miscellaneous News
- Pharmaceutical News
- Women's Health News
- Archived Stories
- Legals

Medical Books

Medical Conferences

Notable Publications

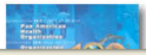

News-Medical.Net

Quotemonster.com - Free Health Insurance Quotes and Information focusing on Individual, Family, Self Employed, and Small Business Health Insurance Plans. Save up to 75% Instantly!
Health Insurance Quotes | Individual Health Insurance | Health Insurance

People who use their computers to find health information often wind up in worse condition than if they had listened to their doctor

Medical Study News
Published: Monday, 18-Oct-2004

Printer Friendly | Email to a Friend

People who use their computers to find information about their chronic disease often wind up in worse condition than if they had listened to their doctor, according to a University College London (UCL) review of studies on internet health.

Using interactive computer tools does improve the medical knowledge of people with diabetes, asthma or other chronic conditions, and does provide them with positive feelings of social support, according to researchers reviewing 28 randomized controlled trials involving 4,042 participants. But there was no evidence that cyber-medicine helps people change their behavior and startling evidence that it may leave them in worse health.

"This whole finding confounds conventional wisdom," says lead author Dr Elizabeth Murray of UCL's Department of Primary Care and Population Sciences. The authors looked at studies that measured the effectiveness of Interactive Health Communication Applications (IHCAs) on people's information gain, feelings of social support, self-efficacy, behavior change and overall clinical outcomes.

IHCAs had a positive effect on people's information gain and feelings of social support; no effect on self-efficacy (the belief that behavior change is possible) or on actual behavior change; and a strikingly negative effect on outcomes.

Dr Murray said there are two possible reasons for the paradox between active knowledge-seekers and their seemingly worsening health.

One reason may be that when they learn of small, but important, statistical effects of a treatment they become less frightened and thus unmotivated to change the way they might if a doctor bluntly told a person with diabetes to control her sugar or face death. "But actually," Murray explains, "if you become more knowledgeable you realize it's all rather a long way off. They are less frightened and that reduces their motivation to be really strict in their control."

A second reason might be because knowledge-seekers become so steeped in information from the Internet they make treatment choices on their own, contradicting advice from their doctors. For instance, a diabetic person might be told by a doctor to lower blood sugar but decide, based on his own interpretation of data, that the short-term tradeoffs of not complying are worth the long-term risks.

"We found that people who use these things (IHCAs) had more sugar in their blood than those who didn't," Dr Murray said.

The researchers cannot explain the finding that the interventions had no effect on behavior change but nevertheless resulted in worse outcomes. Murray and her team conclude that more research is needed to fully understand the negative effect of interactive health applications on clinical outcomes and whether some computer health programs can be designed to improve them.

Ads By Google

Diabetes Research
Manage and treat Diabetes
Prevent complications today!
www.LifeScan.com

Juvenile Diabetes
If you don't know your blood sugar levels, you should. Learn more here
www.Ascensia.ca

Medical News
Get a Canadian perspective with up-to-the-minute reports.
cbc.ca/science

Diabetics - Secrets
The Pharmaceutical Industry Doesn't Want You To Find Out - Free Report
www.throwoutthemeds.com

Treatment Guidelines
ODG Treatment in Workers Comp based on empirical medical evidence
www.disabilitydurations.com

Type 1 Diabetes Info.
Information you can trust on type 1 diabetes and more!
www.diabeteshealthonline.com

Diabetes
Resource on Diabetes.
Focus your Medical Search!
Guide2Med.com

Help for Diabetes is Here
Diabetics Get Lower Blood Sugar in Just 4 Hours. Money Back Guarantee
www.chayacorp.com

Done

start | Desktop | Links | 98% | 6:05 AM Saturday 28/05/2005

BBC NEWS | Health | Warning on internet health advice - Mozilla Firefox
File Edit View Go Bookmarks Tools Help
http://news.bbc.co.uk/2/hi/health/3746056.stm
~AdSense ~blue ~BMJ ~CNN ~dict ~EasyWeb ~Globe ~Google Scholar ~HubMed ~iplanet ~JMIR ~mls.ca ~PayPal ~PubMed ~SPIEGEL TOStar
Open Journal Systems: Sub... | BBC NEWS | Health | W... | EasyWeb | PayPal - My Account | The Beach Tour - Beaches ... | Brochures

Last Updated: Sunday, 17 October, 2004, 23:11 GMT 00:11 UK

E-mail this to a friend   Printable version

Warning on internet health advice

People with chronic disease should think twice before relying on the internet for health advice, research suggests.

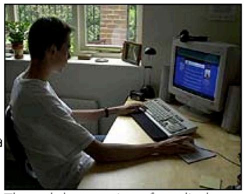

The web has a mine of medical information

A University College London study found many would be in a better condition if they had simply listened to their doctor's advice.

The study found interactive computer tools do improve medical knowledge.

But there was no evidence they change behaviour positively, and some that they left people in worse health.

The UCL team reviewed the effect of interactive web tools - known technically as Interactive Health Communication Applications (IHCAs) - on people with long-term conditions such as diabetes and asthma.

In total they looked at 28 studies covering 4,042 participants.

Lead researcher Dr Elizabeth Murray said she was surprised that the tools seemed to boost medical knowledge, but, if anything, have a negative effect on the users' health.

Paradox

Dr Murray said one reason for this apparent paradox may be that titbits of information may lull users into a false sense of security.

Thus they become less motivated to control their condition in the way they would be if faced with a blunt instruction from a doctor.

Alternatively, users may become so steeped in information that they make treatment choices on their own, contradicting advice from their doctors.

For instance, a diabetic person might be told by a doctor to [illegible]

SEE ALSO:
Rating call for cancer websites
15 Apr 04 | Health
Health websites no threat to GPs
12 May 04 | Health
'The internet saved my life'
06 Oct 03 | Health

RELATED INTERNET LINKS:
University College London
The BBC is not responsible for the content of external internet sites

TOP HEALTH STORIES NOW
Women 'risking health for others'
Sleep disorder liver disease link
US probes Viagra 'blindness risk'
Chemicals may harm male babies
RSS | What is RSS?

Done
start
98%
6:06 AM Saturday 28/05/2005

Sympatico/MSN News - CBC.ca - Mozilla Firefox
File Edit View Go Bookmarks Tools Help
http://sympatico.msn.cbc.ca/story/science/national/2004/10/18/internet_health041018.html

Home
Top Stories
National
World
Business
Sports
Entertainment
Health
Science & Tech
Weather
Lotteries
Horoscopes
Special Features
Unusual News

CBC.CA : NEWS : RADIO : TELEVISION : LOCAL SEARCH

Internet-based health information may be hazardous: study

Last Updated Mon, 18 Oct 2004 14:55:28 EDT
CBC News

LONDON - People with chronic diseases ought to pay more attention to what their doctor tells them rather than relying on advice from the internet, according to a new study.

Researchers at University College London reviewed clinical trials that measured the effectiveness of computer-based tools called Interactive Health Communication Applications.

The tools are usually web-based health packages that combine information with an online support group or chat room.

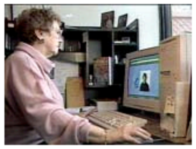

The web may help patients to find social supports but they may not fair as well.

Cyber-medicine improved medical knowledge among people with chronic conditions such as diabetes or asthma, the team found.

But there was no evidence patients changed their behaviour for the better, and some were left in worse health, the researchers found.

One reason for the paradox is patients may be lulled into a false of security online and therefore they're less motivated to act to improve their health.

Or users may rely on the internet information although it contradicts their doctor's advice. For example, a diabetic may decide the long-term risks of not lowering her blood sugar are worth the short-term gains.

"We found that people who use these [tools] had more sugar in their blood than those who didn't," said the study's lead author, Dr. Elizabeth Murray.

Murray's team reviewed 28 randomized controlled trials involving 4,042 participants.

"Consumers who wish to increase their knowledge or social support amongst people with a similar problem may find an IHCA helpful," the researchers concluded. "However, consumers whose primary aim is to achieve optimal clinical outcomes should not use an IHCA at present."

Advertisement
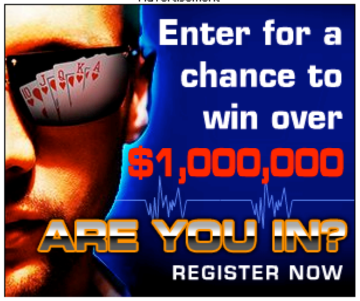

Find: deposit Find Next Find Previous Highlight Match case
Transferring data from sdc.cbc.ca...
start 96% 8:15 AM Monday 30/05/2005

MUSIC backstage
GET THE LATEST • videos • photos • charts • buzz • news
FREE newsletter!
SIGN UP NOW!

News by Source
- News Main Page
- American Cancer Society
- Associated Press
- HealthDay
- KidsHealth.org
- Reuters

More on Yahoo! Health
- Ask the Expert
- Clinical Trials
- Drug Guide
- Encyclopedia A-Z
- Health News via RSS
- Recipes

HealthDay | All HealthDay News

Web Not Always Safe Health Source for Some

HealthDay

MY Y! Yahoo! Health: Asthma News

October 21, 2004 08:40:57 PM PST

THURSDAY, Oct. 21 (HealthDayNews) -- Internet-based health information may be hazardous to people with chronic diseases such as diabetes or asthma, say University College London researchers.

They reviewed 28 studies on health information from the Web and found that using interactive computer tools does help people improve their medical knowledge and provides them with positive feelings of social support.

But the study found no evidence that Web health information helps people with chronic diseases make behavior changes that will benefit them. In fact, people who use computers to access information about their disease often end up in worse condition than if they had listened to their doctor.

"This whole finding confounds conventional wisdom," study author Dr. Elizabeth Murray, of the department of primary care and population sciences, said in a prepared statement.

She said patients who access a lot of Web-based health information may decide to make their own treatment choices, even if those decisions are contrary to a doctor's advice.

For example, a person with diabetes may be told by a doctor to lower

ADVERTISEMENT
MUSIC Watch Music Videos On-Demand!
Nelly Errtime
Akon Lonely
Ying Yang Twins Wait
Jesse McCartney

TIME Magazine Archive Article -- Click To Get Sick? -- Nov. 01, 2004 - Mozilla Firefox

http://www.time.com/time/archive/preview/0,10987,733789,00.html

Find Your Graduating Class

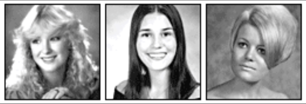

classmates.com

1994 1984 1974 1964 1954

TIME ARCHIVE 1923 to the Present

MAGAZINE CUSTOMER SERVICE
- Renew your subscription
- Send the Gift of TIME
- Change Your Address

Articles | Covers

Search [Search] · Advanced Search · See All Covers · Search Tips

Search From: Jan 1 1923 through Dec 31 2005

YOUR TIME/HEALTH/PAGING DR. GUPTA

Click To Get Sick?

People who get medical advice from the Web actually get worse, a study shows

By SANJAY GUPTA

Print E-Mail Save Popular

>Subscribe to TIME

Nov. 1, 2004

There's a running gag in Floater, Calvin Trillin's 1980 comic novel about a newsmagazine that sounds a lot like TIME, in which the medicine writer comes down with the symptoms of whatever disease he's writing about that week. I was reminded of that hapless writer when I read about a new study out of University College London that found that people who use the Web to get information about their chronic diseases often wind up in worse shape than before they logged on. Could the medical information on the Internet actually be making people ill? Not exactly, says Dr. Elizabeth Murray of the college's...

ALSO IN THIS ISSUE

Nov. 1, 2004

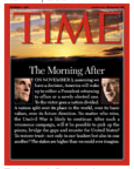

Table of Contents »
Photos and Graphics »

To continue reading the complete article, login or subscribe below and get free instant access. Get 6 issues of TIME for only $1.99

ARCHIVE FEATURES

- **Archive Homepage:** Best of the archive
- **Collections:** Oscar Greats, Love & Sex
- **Browse by Issue:** Click covers to see contents
- **Cover Galleries:** Browse by topic or year
- **Search Tips:** Find the articles you want

ADVERTISEMENT

Find Your Graduating Class

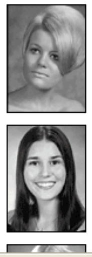

Find: deposit Find Next Find Previous Highlight Match case

Done

8:30 AM Monday 30/05/2005

Aktuelles
Leben mit MS
Was ist MS?
Häufig gestellte Fragen
Medizin und Therapie
Recht
Reise und Freizeit
MS von A-Z
Geschichte der MS
DMSG-Datenbank
Wir über uns
Ansprechpartner
Mitgliedschaft
Spenden und helfen
Service
Termine
Foren
ExpertenChat
Forum
ExpertenChat
Bildergalerien

© AMSEL 2005
Medizinischer Fortschritt und MS: Ursachen, Forschung und Therapie der Multiple Sklerose

MULTIPLE SKLEROSE · MEDIZIN/THERAPIE

So viel Information war nie

20.10.04 - Und doch sind Internetnutzer oft die weniger vernünftigen Patienten. Auch MS-Betroffene sollten wachsam mit dem Medium umgehen.

Wie eine Meta-Analyse der Cochrane Collaboration zeigt, ist der informierte Patient nicht unbedingt der vernünftigere Patient. Das Internet gehört zu den computergestützten Paketen, die idealerweise unter Kontrolle von Gutachtern Gesundheitsinformationen für Patienten anbieten, um diese bei Entscheidungen zu unterstützen *und* nach Möglichkeit eine positive Veränderung des Verhaltens zu bewirken. Solche seriösen Angebote erreichen jedoch nicht immer ihr Ziel, so die Einschätzung von Dr. Elizabeth Murray, die für die Cochrane Collaboration acht randomisierte kontrollierte Studien zur computergesteuerten Gesundheitsinfo in einer Meta-Analyse zusammengefasst hat.

Das Ergebnis scheint zunächst positiv: Im Vergleich zum Arztgespräch sind die übers Netz geschulten Patienten besser über ihre eigene Krankheit informiert. Auch erfahren sie eine gewünschte soziale Unterstützung. Die erhoffte Verhaltensänderung tritt jedoch nicht ein. Schlimmer noch: In einigen Studien verhalten sich diese Patienten weniger vernünftig als Patienten, die ihrem Arzt vertrauen. Vor allem chronisch Kranke laufen Gefahr, vor lauter Informationsflut das eigentliche Ziel aus den Augen zu verlieren.

Dies erläutert Murray am Beispiel eines Diabetikers: Wenn dieser von seinem Arzt eindringlich auf die negativen Folgen einer schlechten Blutzuckereinstellung hingewiesen wird, habe dies häufig eine nachhaltigere Wirkung, als wenn der gleiche Patient sich später im Internet über die wissenschaftlichen Grundlagen des ärztlichen Ratschlags informiert. Dort erfährt er nämlich, dass ein erhöhter Blutzucker kurzzeitig keine negativen Auswirkungen hat, was der Arzt ihm in der verkürzten Darstellung verschwiegen haben mag. Doch wenn [illegible]

www.junge-initiativen.de
Die Plattform für junge MS-Betroffene der AMSEL

Die AMSEL-Kontaktgruppe in Ihrer Nähe

Gesundheitsreform

"Mit dem Gesetz können alle Beteiligten leben." *Ulla Schmidt*

Weitere Artikel zur Gesundheitsreform:
· Krankentransport und Zuzahlungsregelung weiterhin Knackpunkte der Gesundheitsreform
· Nach der Reform - wichtige Infos zusammengefasst
· Chronikerregelung und Krankentransport-Richtlinien verabschiedet
· Gesundheitsreform - Start mit Hindernissen
· Virtuelle Ratgeberin nimmt ihre Arbeit auf
· Ministerium verlangt Ergänzung der Heilmittelrichtlinien
· Kassen zahlen Fahrtkosten für hilfsbedürftige Patienten

Die Geschichte der MS

SvD: Sjuka blir sjukare när de hämtar kunskap på nätet - Mozilla Firefox
http://www.svd.se/dynamiskt/inrikes/did_8307326.asp

Här har du utmaningen! Vilken morgontidning är bäst – SvD eller DN?!

Publicerat 18 oktober 2004 07:00

Sjuka blir sjukare när de hämtar kunskap på nätet

Sjuka personer som använder internet för att få information om sin sjukdom får visserligen mer kunskap, men deras hälsa försämras i stället för att förbättras. Det överraskande resultatet kom fram i den första omfattande studien av vilka effekter de många hälsosajterna har.

Om du är eller blir sjuk så undvik att surfa på de otaliga hälsosajterna på nätet. Du riskerar nämligen att bli sämre i din sjukdom.

Den oväntade rekommendationen kan utläsas i den första undersökningen av vilka effekter det stora hälsoutbudet på internet har på människor med kroniska sjukdomar.

Information om medicin och hälsa är det enskilt största ämnesområdet på internet.

På nätet
Cochrane

Bakom analysen står det prestigefyllda Cochrane, ett internationellt nätverk av forskare som sysslar med att kvalitetsmäta och utvärdera all tillgänglig medicinsk forskning.

I detta fall är det en grupp utvärderare under ledning av forskare vid University College London i Storbritannien som analyserat alla studier av hur internets hälsosajter påverkar sjuka personer.

De hittade 28 olika studier där grupper av sjuka internetanvändare jämförs med andra som inte surfar. Samtliga av de över 4 000 deltagande patienter led av livslånga sjukdomar, främst diabetes och astma.

De aktuella hälsosajterna var interaktiva och höll hög kvalitet. De innehöll alltså inte enbart ren medicinsk information utan gav också möjlighet att ställa frågor till experter och chatta med andra sajtläsare.

Internetanvändarna var som väntat mer informerade om sin sjukdom än andra och de tyckte också att de hade fått ett större socialt nätverk. Men trots detta hade de inte kunnat börja leva mer hälsosamt. Och det mest paradoxala var att internetanvändarna faktiskt blev sämre i sin sjukdom.

Skillnaderna i hälsa jämfört med de sjuka som inte surfade kunde tydligt mätas i till exempel blodsockernivåer.

Forskarna själva är lika förvånade som alla andra och menar att sjukdomsförsämringen är oförklarlig och kräver ytterligare

Tipsa en vän
Skriv ut sidan
Kommentera
Mest läst nu

- Pianomannen - tjeckiskt geni?
- M siktar åter på systemskifte
- För evigt fascinerad av fascismen

Dagens fråga
Ska Sverige fortsätta processen med att ta ställning till det nya EU-fördraget?
Ja
Nej
Se resultatet

Söndagsintervjun

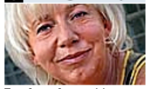

Eva Lundgren blev Rikshäxa efter Könskriget där hon påstår att barn offras i skogen.

Mardröm

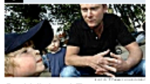

Carl Michael Bergman förlorade sin fru i flodvågskatastrofen. Och mardrömmen fortsätter.

SvD granskar

ANNONS

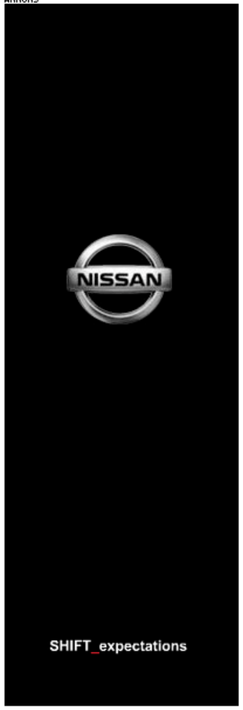

SvD Internet sök

ANNONS
Sök
Företag/person/nr
Adress/plats
hitta.se sök

blocket
Bilar 103450
Båtar 9389
MC 15985
Bostad 15553
Möbler 34106
Övrigt 162178
Annonsera

PRENUMERERA
ANNONSERA

SvD UTMANINGEN
Testa SvD 1 månad 98:-
Klicka här

Snabblänkar A-Ö
- Aktier
- Berglin
- Bilar
- Bildspecial
- Bokrecensioner
- Bragdguldet
- Brännpunkt
- Börs&finans

Find: deposit Find Next Find Previous Highlight Match case
Done
7:44 AM Monday 30/05/2005

Press Release Archive - Microsoft Internet Explorer

File Edit View Favorites Tools Help

Address C:\privat\TEXTE\PUB\jmir-editorials\2004-cochrane\media\Press Release Archive.htm

!Please Note!

The review "Interactive Health Communication Applications for people with chronic disease" by Elizabeth Murray, Jo Burns, Sharon See Tai, Rosalind Lai, Irwin Nazareth, published on issue 4 2004 of The Cochrane Library , has been found to contain an error. This meant the direction of change was incorrect for several clinical and behavioural outcomes.

The authors and editorial team have responded immediately, and will be withdrawing the review from The Cochrane Library for revision to correct the error. The authors and editorial team apologise to all concerned.

An official statement has been posted here: http://www.mrw.interscience.wiley.com/cochrane/clsysrev/articles/CD004274/frame.html

Please do not use information from the following release until the review has been re-analysed

18 October 2004

Knowledge may be hazardous to web consumers' health

People who use their computers to find information about their chronic disease often wind up in worse condition than if they had listened to their doctor, according to a University College London (UCL) review of studies on internet health.

Using interactive computer tools does improve the medical knowledge of people with diabetes, asthma or other chronic conditions, and does provide them with positive feelings of social support, according to researchers reviewing 28 randomized controlled trials involving 4,042 participants. But there was no evidence that cyber-medicine helps people change their behavior and startling evidence that it may leave them in worse health.

"This whole finding confounds conventional wisdom," says lead author Dr Elizabeth Murray of UCL's Department of Primary Care and Population Sciences. The authors looked at studies that measured the effectiveness of Interactive Health Communication Applications (IHCAs) on people's information gain, feelings of social support, self-efficacy, behavior change and overall clinical outcomes.

IHCAs had a positive effect on people's information gain and feelings of social support; no effect on self-efficacy (the belief that behavior change is possible) or on actual behavior change; and a strikingly negative effect on outcomes.

Dr Murray said there are two possible reasons for the paradox between active knowledge-seekers and their seemingly worsening health.

One reason may be that when they learn of small, but important, statistical effects of a treatment they become less frightened and thus unmotivated to change the way they might if a doctor bluntly told a person with diabetes to control her sugar or face death. "But actually," Murray explains, "if you become more knowledgeable you realize it's all rather a long way off. They are less frightened and that reduces their motivation to be really strict in their control."

A second reason might be because knowledge-seekers become so steeped in information from the Internet they make treatment choices on their own, contradicting advice from their doctors. For instance, a diabetic person might be told by a doctor to lower blood sugar but decide, based on his own interpretation of data, that the short-term tradeoffs of not complying are worth the long-term risks.

Contact

Dominique Fourniol
Head of Media Relations
+44 (0)20 7679 9728

Jenny Gimpel
Media Relations Manager
+44 (0)20 7679 9739

Alex Brew
Media Relations Manager
+44 (0)20 7679 9726

Post vacant
Media Relations Manager
+44 (0)20 7679 7678

Out of hours:
07917271364

Error on page.

24 of 24 - Clipboard
Item collected.

7:08 AM Monday 30/05/2005

Too much advice can be bad for your health - Everybody - Health Information for New Zealanders - Mozilla Firefox

File Edit View Go Bookmarks Tools Help

http://www.everybody.co.nz/page-c816e3c8-1f1f-4a0f-8b1a-5a6c6f468f82.aspx Go elizabeth murray corch

~AdSense ~blue ~BMJ ~CNN ~dict ~EasyWeb ~Globe ~Google Scholar ~HubMed ~iplanet ~JMIR ~mls.ca ~PayPal ~PubMed ~SPIEGEL TOStar »

Open Journal Systems: Sub... | The Globe and Mail: France ... | EasyWeb | PayPal - My Account | Google Search: breach an a... | Too much advice can be ...

home | about us | bulletin board | tools | contact | Search | tell a friend | print

everybody

BreastScreen AOTEAROA
FREEPHONE 0800 270 200 • www.healthywomen.org.nz
Advertisers do not influence the editorial content of this page.

Support Groups | Healthy Living in NZ | Tests & Treatments | Health Research | Nutrition Research | OTC Medicines Guide | Quizzes

Health Centres

- Allergy
- Arthritis
- Asthma
- Cancer
- Colds and 'Flu
- Continence
- Diabetes
- Eye
- Gastrointestinal
- Heart
- Men's Health
- Mental Health
- Nutrition & Weight
- Osteoporosis
- Pain
- Parenting
- Pregnancy
- Senior's Health
- Sexual Health
- Sports & Fitness
- Surgery
- Tests & Treatments
- Travel
- Women's Health

Bulletin Board

Questions, Answers & Communities

Looking For?

- a LMC
- a Hospital
- a Travel Doctor

Looking For?

Health Topics A-Z Quick Links

TOO MUCH ADVICE CAN BE BAD FOR YOUR HEALTH

Some people with chronic health problems who seek online advice would be better off just listening to their doctors, new research suggests. While advice provided over the Internet generally helps patients improve medical knowledge, it doesn't do much to actually improve their health, according to scientists at University College London. In fact, some people who relied mostly on the online advice were found to be in poorer health than those who were mostly counseled by their doctors. Lead researcher Dr Elizabeth Murray suggested that advice found online might lead some people to make treatment choices on their own, contradicting recommendations from their doctors, reported BBC News Online. Internet-based advice also tends to be less dramatic and frightening, perhaps lessening patients' feelings of urgency, Murray speculated. Her study involving more than 4000 patients is published by the Cochrane Collaboration, an international organisation that evaluates medical research. (Health Scout)

Back to the News Archives index

top

Tools

WHAT'S YOUR BMI?
Calculate your Body Mass Index (BMI).

BABY'S DUE DATE
Find out your baby's due date.

HEART ATTACK AND STROKE RISK QUIZ
Try our quiz on heart attack and stroke risk - it affects us all (no one is totally risk-free). Your doctor or nurse will be happy to sit down and talk through ways you can reduce your health risks.

SMOKING COST CALCULATOR
Find out how much you could save in a year by quitting smoking here.

HEART RATE FOR FITNESS
To work out your fitness zone heart rate click here.

MEDICAL DICTIONARY
To find the meaning of

Find: deposit | Find Next | Find Previous | Highlight | Match case

Done

start | Desktop | Links | 96% | 7:27 AM Monday 30/05/2005

NewsFromRussia.Com Beware of Internet health advises - Mozilla Firefox

File Edit View Go Bookmarks Tools Help

http://newsfromrussia.com/science/2004/10/18/56669.html Go quotes irreversible dan

~AdSense ~blue ~BMJ ~CNN ~dict ~EasyWeb ~Globe ~Google Scholar ~HubMed ~iplanet ~JMIR ~mls.ca ~PayPal ~PubMed ~SPIEGEL TOStar »

Bowker's Books In Print... | Open Journal Systems:... | Google Search: quotes ... | Open Journal Systems:... | Journal of Medical Inter... | NewsFromRussia.C... | Google Search: Global ...

news from russia

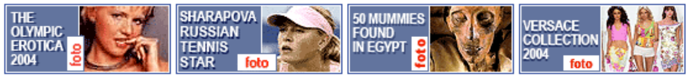

pravda.ru sites ...

Pravda.RU:Science and Culture:More in detail

Beware of Internet health advises

18:59 2004-10-18

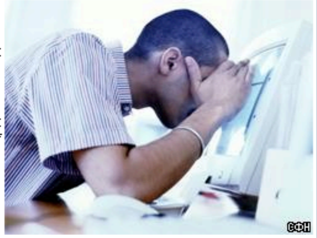

According to new British study, patients who use the internet to find out information about chronic disease rather than listening to their doctor could be putting their health at risk.

Using interactive computer tools, such as online support groups and chatrooms, does help improve the knowledge of people with conditions such as asthma and diabetes and provides social support.

But researchers from University College London said there was no evidence that "cyber-medicine" helped people change their behaviour.

They said there was evidence using interactive services may actually leave patients in worse health, reports informs The Scotsman.

Suppose 1,000 patients who take their medicine reduce the number of heart attacks for their group by half a dozen. Doctors will say this proves patients should take their medicine, she said.

But a patient may see this the other way around. The financial cost and perhaps side-effects of the medication might be too great when the chances are greater than 99 per cent that it won't bring him or her any benefit.

"You might say, 'Do I want the unpleasantness of taking medicine for a small risk reduction?' " she said. If this is an informed decision, then it may make sense for the patient.

"It's important not to see this negative clinical outcome as necessarily negative from the patient's point of view," informs Canada.com.

Dr. Murray said there are two possible reasons for the paradox between active knowledge-seekers and their seemingly worsening health. One, that when they learn of small, but important, statistical effects of a treatment they become less frightened and thus unmotivated to change the way they might if a doctor bluntly told a person with diabetes to control her sugar or face death.Two, knowledge-seekers become so steeped in information from the Internet they make treatment choices on their own, contradicting advice from their doctors.

"But actually,if you become more knowledgeable you realize it's all rather a long way off. They are less frightened and that reduces their motivation to be really strict in their control. We found that people who use these things (IHCAs) had more sugar in their blood than those who didn't," Murray said, informs New Kerala.

Calendar

Mo	Tu	We	Th	Fr	Sa	Su
				1	2	3
4	5	6	7	8	9	10
11	12	13	14	15	16	17
18	19	20	21	22	23	24
25	26	27	28	29	30	31

Sep/2004 Nov/2004

Science and Health Analytical news

- Written language of the Maya shocks scientists with its brutality
- Special unit of Russian aviation uses cement particles and acids to change weather
- Russian-US solar sail satellite project fails
- Nightingales to sing at the International Space Station
- Russian settlement still reveals traces of dinosaurs
- Specialists find scientific explanation to phenomenon of living dead
- Sedatives have different effect on early risers and late-night creatures
- NASA spaceprobe to search for water in space using Russian technology
- Archeologists uncover cursed Prussian treasure in Kaliningrad's Royal

Done

start Desktop Links 100% 8:07 PM Monday 27/06/2005

Concession season tickets – from under £6 a match

NEWS.scotsman.com
Scottish news direct from Scotland
Tuesday, 28th June 2005

Health

Evening News
Mon 18 Oct 2004

printer friendly | email article

Fears over health 'cures' on the web

LYNDSAY MOSS

PATIENTS who use the internet to find out information about chronic disease rather than listening to their doctor could be putting their health at risk, research suggested today.

Using interactive computer tools, such as online support groups and chatrooms, does help improve the knowledge of people with conditions such as asthma and diabetes and provides social support.

But researchers from University College London said there was no evidence that "cyber-medicine" helped people change their behaviour.

They said there was evidence using interactive services may actually leave patients in worse health.

The team, led by Dr Elizabeth Murray, reviewed 28 trials involving more than 4000 participants to measure the effectiveness of Interactive Health Communication Applications (IHCAs).

These communication applications were defined as computer-based information sources combined with other services, like online support groups, chatrooms or advice. The researchers found the IHCAs had a positive effect on people gaining information and feelings of support.

But they concluded the applications had no effect on making patients believe changing their behaviour for the better was possible or on resulting in behaviour change.

They also said they had a "strikingly negative effect" on health outcomes, leaving some in worse health.

Dr Murray said one reason for patients' worse health was that when they learn of small but important statistical effects of their treatment they become less frightened.

This would leave them less motivated to change the way they behave than if a doctor bluntly told a diabetic to control their sugar level or face death, for example.

Dr Murray also said that "knowledge-seekers" might become "so steeped in information" from the internet that they make their own decisions.

PERSONAL FINANCE
Plenty of sensible advice on making your money work for you
SCOTSMAN.COM PREMIUM

NEWS
BUSINESS
SPORTS
ARTS & ENTERTAINMENT
WEATHER
HEALTH & SCIENCE
CBC ARCHIVES
KIDS
TEENS
PROGRAM GUIDE
E-MAIL NEWSLETTERS
SERVICES
CONTACT US
ABOUT CBC
RADIO-CANADA
shop

Internet-based health information may be hazardous: study

Last Updated Mon, 18 Oct 2004 14:55:28 EDT
CBC News

LONDON - People with chronic diseases ought to pay more attention to what their doctor tells them rather than relying on advice from the internet, according to a new study.

Researchers at University College London reviewed clinical trials that measured the effectiveness of computer-based tools called Interactive Health Communication Applications.

The tools are usually web-based health packages that combine information with an online support group or chat room.

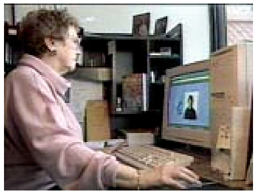

The web may help patients to find social supports but they may not fair as well.

Cyber-medicine improved medical knowledge among people with chronic conditions such as diabetes or asthma, the team found.

But there was no evidence patients changed their behaviour for the better, and some were left in worse health, the researchers found.

One reason for the paradox is patients may be lulled into a false of security online and therefore they're less motivated to act to improve their health.

Or users may rely on the internet information although it contradicts their doctor's advice. For example, a diabetic may decide the long-term risks of not lowering her blood sugar are worth the short-term gains.

EXTERNAL LINKS

- Abstract of review, Cochrane Collaboration

(Note: CBC does not endorse and is not responsible for the content of external sites - links will open in new window)

FEEDBACK
Send your feedback
Read your letters
Report a typo or inaccuracy

TOOLS
Printable version
E-mail this story

Done

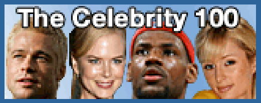

Health

Web Not Always Safe Health Source for Some

-- Robert Preidt

THURSDAY, Oct. 21 (HealthDayNews) -- Internet-based health information may be hazardous to people with chronic diseases such as diabetes or asthma, say University College London researchers.

They reviewed 28 studies on health information from the Web and found that using interactive computer tools does help people improve their medical knowledge and provides them with positive feelings of social support.

But the study found no evidence that Web health information helps people with chronic diseases make behavior changes that will benefit them. In fact, people who use computers to access information about their disease often end up in worse condition than if they had listened to their doctor.

"This whole finding confounds conventional wisdom," study author Dr. Elizabeth Murray, of the department of primary care and population sciences, said in a prepared statement.

She said patients who access a lot of Web-based health information may decide to make their own treatment choices, even if those decisions are contrary to a doctor's advice.

For example, a person with diabetes may be told by a doctor to lower blood sugar. But, based on his or her own interpretation of data from the Internet, the patient may decide the short-term tradeoffs of not

ADVERTISEMENT

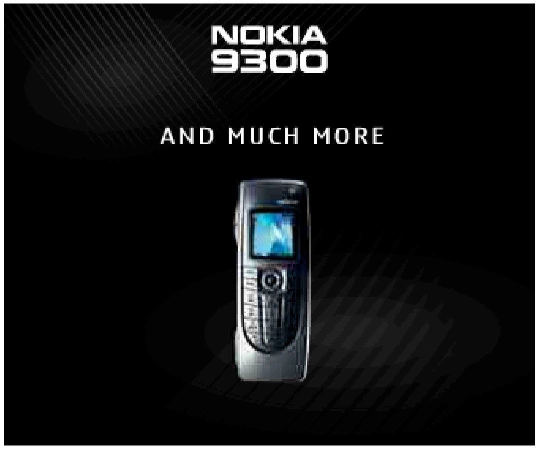

Free Trial Issue of *Forbes* | Gift Subscriptions

E-Mail Alerts
Get stories by e-mail on this topic. FREE
Topics
HealthDay-CMPU
HealthDay-Cat-HealthTech
HealthDay-DIAB
HealthDay-Cat-Diabetes
Enter E-Mail Address: FAQ | Privacy Policy
Sign Me Up!
E-MAIL | DESKTOP

Refinance Now!
Click Your Current Mortgage Rate
Under 5.99% 6.00-6.99%
7.00-7.99% Over 8.00%
LowerMyBills.com
©2004 LowerMyBills.com Inc. All rights reserved.

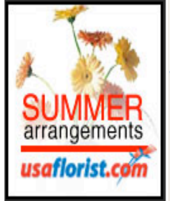

We Have Recently Made Changes to Our Website, If you are unable to find something Specific, Please Search Below

Google
Search
Web onlypunjab.com

Ads by Google

Salwar
Fantastic prices on Salwar Millions of items available daily
www.ebay.ca

Salwar Kameez
Quality new and used items. Find salwar kameez now!
www.ebay.com

Shahnaz Husain Skincare
Shahnaz Husain: Herbal Beauty-care. Anti-aging, Pigmentation Treatment.
www.tofah.com

Bollywood Party Clothing
Salwars, Churidars, Trouser Suits Designer Indian Outfits
Cbazaar.com

Warning on internet health advice

Publish Date : 10/18/2004 12:17:00 PM Source : Health News Onlypunjab.com

People with chronic disease should think twice before relying on the internet for health advice, research suggests.

A University College London study found many would be in a better condition if they had simply listened to their doctor's advice.

The study found interactive computer tools do improve medical knowledge.

But there was no evidence they change behaviour positively, and some that they left people in worse health.

The UCL team reviewed the effect of interactive web tools - known technically as Interactive Health Communication Applications (IHCAs) - on people with long-term conditions such as diabetes and asthma.

In total they looked at 28 studies covering 4,042 participants.

Lead researcher Dr Elizabeth Murray said she was surprised that the tools seemed to boost medical knowledge, but, if anything, have a negative effect on the users' health.

Paradox

Study: Internet medical advice risky - Microsoft Internet Explorer
File Edit View Favorites Tools Help
BigNewsNetwork.com
News Search
GO
Breaking News Business Entertainment International Internet Sports Technology Discuss the News
Home/
Home
Breaking News
Business
Editorials
Entertainment
Health
International
Internet
Markets
Media
Religion
Special Interest
Sports
Technology

Monday 27th June, 2005

Study: Internet medical advice risky

Big News Network.com Monday 18th October, 2004 (UPI)

Those who use the Internet to research their own chronic diseases often wind up in worse condition than if they had heeded a doctor's advice.

A review of 28 randomized controlled trials involving 4,042 participants by lead author Dr. Elizabeth Murray of the University College Medical School in London found using computer tools does improve the medical knowledge of people with diabetes, asthma or other chronic conditions. It also provides them with positive feelings of social support, but there was no evidence cyber-medicine help people change their behavior.

The study suggested because knowledge-seekers become so steeped in information from the Internet, they make treatment choices on their own, contradicting advice from their doctors. For instance, a diabetic person might be told by a doctor to lower blood sugar but decide, based on his own interpretation of data, that the short-term tradeoffs of not complying are worth the long-term risks.

We found that people who use these things (Internet sources) had more sugar in their blood than those who didn't, Murray said.

The review appears in the October issue of the Cochrane Collaboration, an international organization that evaluates medical research.

Print | Email to a friend

Logging on can make you sicker

Judy Skatssoon
ABC Science Online

Wednesday, 20 October 2004

Using the internet to manage a chronic illness can leave you in worse health than before you logged on, a review of recent studies has found.

The University College London (UCL) review of the effect of computer and web-based health programs is published in the latest issue of the Cochrane reviews.

The review looked at children and adults with a range of chronic conditions including diabetes, arthritis, eating disorders, asthma, cancer and Alzheimer's disease.

After looking at 28 trials involving more than 4000 patients, it found that using the internet improved knowledge and gave patients a sense of social support.

But web-based programs had no effect on behaviour and didn't encourage people to change their lifestyle. They also had a negative effect on long-term health.

"Consumers who wish to increase their knowledge or social support amongst people with a similar problem may find [using the internet] helpful," the review said.

"However, consumers whose primary aim is to achieve optimal clinical outcomes should not use [it]."

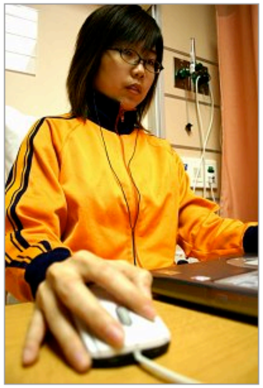

Using the internet to research your medical condition may backfire, a new review suggests *(Image: iStockphoto)*

Should we abandon the net?

Australian researcher Professor Richard Wootton, director of research at the Centre for Online Health at the University of Queensland, said people shouldn't rush to the conclusion that turning to the internet in response to illness was a health hazard.

He said researchers in Queensland and Canberra had shown the internet was valuable in delivering cognitive behaviour therapy for conditions like non-clinical depression and obsessive-compulsive disorder, areas excluded from the Cochrane review.

"I think it's a very interesting finding in a new and potentially important field but it's hard to interpret what it means at this rather early stage of the game," he said.

"It may be that when you get larger studies and more of them this [negative effect] may disappear."

Lead researcher Dr Elizabeth Murray of UCL described the finding as "unexpected and provocative".

Murray said more research was needed to establish whether the negative health outcomes were because patients became over-assured, thereby losing the motivation to change bad habits, or made a decision to prioritise short-

All Science News
Health & Medical
Environment & Nature
Space & Astronomy
Ancient Worlds
Innovation & Technology

Latest News

Rubber tweaked for low-allergy condoms
Computer screen made from fog
Did you spill that oil?

Search News in Science

Search

News Archive

Previous news stories

Science Features

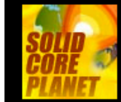

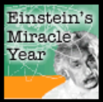

Science by Topic

Ancient Worlds
DNA & Genetics
Sex & Relationships
>> More topics

Email List

first name

surname

Digital Media Europe: News - Patient, don't try to heal thyself - Microsoft Internet Explorer

File Edit View Favorites Tools Help

DMeurope.com
Digital media news for europe

About Us | Syndication | Advertise | Contact Us | Write for DM Europe

Advanced search

Search Go

GSM-Top 10 Articles
Mobile comm news & resources picked by IT experts; Free weekly updates.

Free GSM Wireless Papers
Find White Papers and Reports Download Free Information Here.

Rogers Wireless Phones
Get 6 Months of Unlimited Local Calling. Free Color Camera Phones

Jump-start your career:
Electronics and telecom training at Vancouver Community College

Ads by Google

Internet & e-commerce | itv, DTV, Streaming | Internet Access | Mobile and Wireless | Telecoms | Tech & IT

News by Country

Select country

News by Contributor

Select contributor

Features & Editorials

- Writing for accessibility on the web
- Wi-fi: free and easy
- Alternative, smaller news outlets slam Google News 'qualitative' news gathering system

more »

Week's Top Stories

- Sky to offer movie, sport downloads

United Kingdom > Internet & e-commerce

Patient, don't try to heal thyself

19/10/2004 by John Tilak

Print | Email Colleague | Add Comment | Comments (0)

Looking to the internet for medical help could cause more harm than good, according to a University College London study.

Ironically it is the very role that interactive tools play in enhancing medical knowledge that is the problem.

The study notes that this increase in knowledge could be bad for users by making them more complacent. The availability of information could also prompt them to make their own decisions for their treatment instead of complying with a doctor's prescription.

The review appears in the October issue of the Cochrane Collaboration, an international organisation that evaluates medical research.

Print | Email Colleague | Add Comment | Comments (0)

About

John Tilak

More>> Articles>> Email>>

Related Sites:

- University College London

Free Newsletters

- Daily DMeurope
- Weekly Roundup

Please enter email address

Go

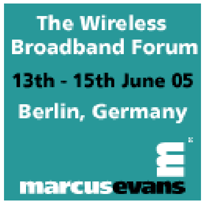

Classified Ads

- **Fixed Mobile Convergence**
From the producers of the world renowned 3GSM World Congress, IBC Telecoms Conferences are proud to present FMC. Discuss the latest developments with International Fixed and Mobile Operators: BT, Vodacom,

© Newswise.
All Rights Reserved.

Source: Health Behavior News Service

Released: Wed 13-Oct-2004, 09:10 ET
Embargo expired: Sun 17-Oct-2004, 19:00 ET
Printer-friendly Version

Knowledge May be Hazardous to Web Consumers' Health

Libraries
Life News (Social and Behavioral Sciences)

Keywords
INTERNET, HEALTH, WEB, ONLINE, MEDICAL, DIAGNOSIS, DISEASE

Contact Information
Available for logged-in reporters only

Description
People who use their computers to find information about their chronic disease often wind up in worse condition than if they had listened to their doctor, according to a new review of studies on Internet health.

Newswise — People who use their computers to find information about their chronic disease often wind up in worse condition than if they had listened to their doctor, according to a new review of studies on Internet health.

Using interactive computer tools does improve the medical knowledge of people with diabetes, asthma or other chronic conditions, and does provide them with positive feelings of social support, according to researchers reviewing 28 randomized controlled trials involving 4,042 participants. But there was no evidence that cyber-medicine help people change their behavior and startling evidence that it may leave them in worse health.

"This whole finding confounds conventional wisdom," says lead author Elizabeth Murray, M.D., Ph.D., of the University College Medical School in London. The authors looked at studies that measured the effectiveness of "Interactive Health Communication Applications" on people's information gain, feelings of social support, "self-efficacy," behavior change and overall clinical outcomes.

IHCAs had a positive effect on people's information gain and feelings of social support; no effect on "self-efficacy" (the belief that behavior change is possible") or on actual behavior change; and a strikingly negative effect on outcomes.

Murray said there are two possible reasons for the paradox between active knowledge-seekers and their seemingly worsening health.

One reason may be that when they learn of small, but important, statistical effects of a treatment they become less frightened and thus unmotivated to change the way they might if a doctor bluntly told a person with diabetes to control her sugar or face death. "But actually," Murray explains, "if you become more knowledgeable you realize it's all rather a long way off. They are less frightened and that reduces their motivation to be really strict in their control."

A second reason might be because knowledge-seekers become so steeped in information from the Internet they make treatment choices on their own, contradicting advice from their doctors. For instance, a diabetic person might be told by a doctor to lower blood sugar but decide, based on his own interpretation of data, that the short-term tradeoffs of not complying are worth the long-term risks.

"We found that people who use these things (IHCAs) had more sugar in their blood than those who didn't," Murray said.

The researchers cannot explain the finding that the interventions had no effect on behavior change but nevertheless resulted in worse outcomes. Murray and her team conclude that more research is needed to fully understand the negative effect of interactive health applications on clinical outcomes and whether some computer health programs can be designed to improve them.

The review appears in the October issue of the *Cochrane Collaboration*, an international organization that evaluates medical research. Systematic reviews draw evidence-based conclusions about medical practice after considering both the content and quality of existing medical trials on a topic.

In this review, the definition of an "interactive health communication application" was a computer-based information source combined with one or more additional services, such as an on-line support group, chat room or tailored advice based on data provided by the user. Murray says, however, that some researchers "worry that the friends you make on computer are not right sort of friends, won't be there for you and

Interactive Health Communication Applications for people with chronic disease - Microsoft Internet Explorer

File Edit View Favorites Tools Help

Address http://www.mrw.interscience.wiley.com/cochrane/clsysrev/articles/CD004274/frame.html

My Profile | Log In

WILEY InterScience

HOME | ABOUT US | CONTACT US | HELP

Home / Medicine and Healthcare / Medicine (general)

The Cochrane Library 2005, Issue 2

right ©2005 by John Wiley & Sons, Inc. All Rights Reserved.

BROWSE ARTICLES BY

Cochrane Reviews | DARE | CENTRAL | Methodology Reviews | Methodology Register | HTA | NHS EED | About | Topics

SEARCH IN THIS TITLE

Go

Cochrane Advanced Search

Search History

[Review]
Interactive Health Communication Applications for people with chronic disease

PDF (Size 117K)

- Reason for Withdrawal
- Sources of support
- Cover sheet

Wiley InterScience is unable to give you access to this content.

Interactive Health Communication Applications for people with chronic disease
The Cochrane Database of Systematic Reviews
E Murray, J Burns, Tai S See, R Lai, I Nazareth

REASON: You are accessing the system as a Guest. As a Guest, you have access to Abstracts-but not Abstracts with References, Graphical Table of Contents or Full Text Articles.

- To view or edit your location details, go to User Info.
- Questions? See Access Explained.
- If you have a license to access this content as a Personal Subscriber, Society Member or through a Trial Offer, login below.
- **NOTE:** This article is also available to registered users via Pay-Per-View. Login below to purchase this article.

Previous versions of this article are available and are listed below. Purchasing this article via Pay-Per-View will give you access to all versions for a 24 hour period.

- Version 2 Published Online: 18 Oct 2004
- Version 1 Published Online: 22 Apr 2003

Registered Users

Username:

Password:

Remember Me | Login

Register Now | Athens Login

Forgot My Password

New Users

New users can register to purchase 24-hour access to this article.

24-Hour Online Access to article US$ 25.00 *

*Sales tax will be applied in Canada.

Submit Registration

About Wiley InterScience | About Wiley | Privacy | Terms & Conditions

Internet

7:07 AM Monday 30/05/2005
